# Workflow-driven catalytic modulation from single-atom catalysts to Au–alloy clusters on graphene

**DOI:** 10.1038/s41598-025-85891-6

**Published:** 2025-01-14

**Authors:** Gabriel Reynald Da Silva, João Paulo Cerqueira Felix, Celso R. C. Rêgo, Alexandre C. Dias, Carlos Maciel de O. Bastos, Maurício J. Piotrowski, Diego Guedes-Sobrinho

**Affiliations:** 1 Department of Chemistry, Federal University of Paraná, Curitiba, 81531-980 Brazil; 2 Institute of Physics “Armando Dias Tavares”, Rio de Janeiro, 20550-900 Brazil; 3 Department of Physics, Federal University of Pelotas, Pelotas, 96010-900 Brazil; 4 Institute of Physics and International Center of Physics, University of Brasília, Brasília, 70919-970 Brazil; 5 Karlsruhe Institute of Technology, Institute of Nanotechnology Hermann-von-Helmholtz-latz, 76021 Karlsruhe, Germany

**Keywords:** Graphene, Cluster alloys, Density functional theory, Workflow, Chemistry, Energy science and technology, Engineering, Materials science, Nanoscience and technology, Physics

## Abstract

Gold-based (Au) nanostructures are efficient catalysts for CO oxidation, hydrogen evolution (HER), and oxygen evolution (OER) reactions, but stabilizing them on graphene (Gr) is challenging due to weak affinity from delocalized $p_{z}$ carbon orbitals. This study investigates forming metal alloys to enhance stability and catalytic performance of Au-based nanocatalysts. Using *ab initio* density functional theory, we characterize ${\text {M}_{(n-x)}\text {Au}_{x}}$ sub-nanoclusters (M = Ni, Pd, Pt, Cu, and Ag) with atomicities $n=1-4$, both in gas-phase and supported on Gr. We find that M atoms act as “anchors,” enhancing binding to Gr and modulating catalytic efficiency. Notably, ${\text {Pt}_{(n-x)}\text {Au}_{x}}$/Gr shows improved stability, with segregation tendencies mitigated upon adsorption on Gr. The *d*-band center ($\varepsilon _{\text {d}}$) model indicates catalytic potential, correlating an optimal $\varepsilon _{\text {d}}$ range of $-1 \text { to }-2$ eV for HER and OER catalysts. Incorporating Au into ${\text{M}_n}$ adjusts $\varepsilon _{\text {d}}$ closer to the Fermi level, especially for Group-10 alloys, offering designs with improved stability and efficiency comparable to pure Au nanocatalysts. Our methodology leveraged SimStack, a workflow framework enabling modeling and analysis, enhancing reproducibility, and accelerating discovery. This work demonstrates SimStack’s pivotal role in advancing the understanding of composition-dependent stability and catalytic properties of Au-alloy clusters, providing a systematic approach to optimize metal-support interactions in catalytic applications.

## Introduction

Gold-based (Au) nanocatalysts remain highly sought after not only for their well-known efficiency in CO oxidation^1^, ${\text{CO}_2}$ reduction^2^, and ${\text{N}_2}$ reduction^3^, but also for their recent demonstration of exceptional catalytic potential for ${\text{H}_2}$ production, especially in hydrogen evolution reactions (HER)^4^, oxygen evolution reactions (OER)^5^, and oxygen reduction reactions (ORR)^6^. The progressive reduction in catalyst dimensionality from nanoparticles $\rightarrow$ nanoclusters $\rightarrow$ clusters, and ultimately single-atom catalysts (SACs)^7,8^, modifies their electronic structure by exposing more active sites^9^, which enhances their catalytic potential. Moreover, the size regime of few-atom clusters exhibit remarkable structural adaptability upon substrate and reactant binding, with potential-dependent fluxionality reshaping and shifting the activity volcano compared to metal surfaces.^10^ However, this increased activity comes with instability in reactive environments, where small Au clusters are prone to sintering, leading to diminished catalytic performance^11^. This urgently needs theoretical design protocols to overcome these stability challenges in Au-based nanocatalysts.

Adsorbing nanocatalysts onto 2D substrates is a well-established strategy in heterogeneous catalysis, facilitating the separation of reaction products from the catalyst in practical applications^12–15^. Graphene (Gr), a cost-effective 2D substrate, is frequently used for anchoring nanocatalysts^16–20^. However, from a design perspective, the use of pristine graphene is often favored because the introduction of defects, such as vacancies or heteroatoms (e.g., oxygen in Gr oxide), can compromise the nanocatalyst’s performance and stability^21–26^. Although several experimental techniques have produced a range of adsorbed systems, including SACs^27–29^, cluster^30–32^, and nanoparticles^33,34^, the empirical design of sub-nanometer catalysts remains nontrivial due to the complexity of achieving reproducible and predictable catalytic performance.

While previous studies have demonstrated that incorporating a second transition metal (TM) into TM nanoclusters can enhance stability and catalytic activity^35–39^, the impact of the 2D support on the physical and chemical properties of these nanoalloys remains under-explored. For instance, it has been shown that introducing an Au atom into Ag clusters (e.g., forming ${\text {Ag}_{12}\text {Au}}$) improves hydrogen affinity and lowers the energy barrier for HER^40^. According to the Sabatier’s principle^41^, optimal catalytic efficiency requires intermediate-strength interactions between the catalyst and adsorbates, ensuring both effective adsorption of reactants and desorption of products. The binding energy thus serves as a key descriptor for evaluating catalytic performance^42–45^.

Despite this, many theoretical studies overlook the influence of the 2D surface on the physical and chemical properties of nanoalloys, particularly its role in stabilizing and enhancing the catalytic performance of nanoclusters^46–49^. For example, while it is known that incorporating Pd into Au clusters can significantly improve adsorptive stability on Gr^50^, the precise role of Gr in modifying the electronic and reactive properties of such catalysts is unclear.

The weak interaction between Au and Gr results in poor adsorptive stability^51^, which limits the potential of these systems. To overcome this while preserving the pristine nature of Gr without chemical modifications such as functionalization^52^, nitrogen doping^53^, or vacancy creation^54^, we propose the incorporation of TMs from Groups 10 and 11 as anchoring agents in Au nanostructures. This approach aims to enhance the stability of nanoalloys by improving their adsorption on Gr without introducing the complexities associated with substrate modifications that can impact the stability and reactivity of bimetallic clusters.

In this study, we employ an automated scientific workflow developed within the SimStack framework to manage the simulation protocol of the binary ${\text {M}_{(n-x)}\text {Au}_x}$ clusters (for M = Ni, Pd, Pt, Cu, and Ag) on graphene substrates. Our workflow facilitated the calculations of structural stability, adsorption energies, and catalytic properties in the gas phase and when adsorbed on graphene. Automating submission, monitoring, and data retrieval processes saves time. It accelerated raw data acquisition, making the project more efficient, as shown in several other works applied in different fields where they chose Simstack as a workflow framework^55–59^. Beyond that, the workflow ensured the reproducibility of our simulations by standardizing computational procedures. This approach underscores the importance of using a workflow framework to handle sophisticated simulation protocols. Our study demonstrates the utility of SimStack as a valuable tool for theorists and experimentalists, revealing the impact of composition and substrate interactions on these nanoalloys’ stability and catalytic potential. Furthermore, we provide a Colab-based notebook that showcases the analysis of our raw data output, enabling users to delve into the physics of these complex systems. Here, we examined binary ${\text {M}_{(n-x)}\text {Au}_x}$ clusters (for M = Ni, Pd, Pt, Cu, and Ag) with atomicities $n = 1\text { to }4$ and compositions $0<x<n$. We also compared unary ${\text {M}_n}$ and ${\text {Au}_n}$ clusters. These systems were modeled both in the gas phase (vacuum - vac.) and adsorbed on graphene (ads.) to evaluate the effects of the 2D substrate on their stability and catalytic potential.

## Results

All alloy configurations, including pGMCs and their less stable isomers for atomicity $n > 2$, in the gas phase and adsorbed on graphene, are available in the Supporting Information. The relative energies for each atomicity *n* are listed to highlight potential degeneracies. For atomicity at $n = 3$, it is worth noting that the adsorption site on graphene is not restricted to a single atom; the adsorption may involve two or even three atoms. In this context, we defined the number of metal atoms bonded to Gr as the number of contact points (see Methods). Understanding the surface site configuration is crucial, especially for the smallest unary and binary clusters. Single-atom adsorption systems are exciting in this regard, providing well-known examples of SACs.

**Figure 1:**
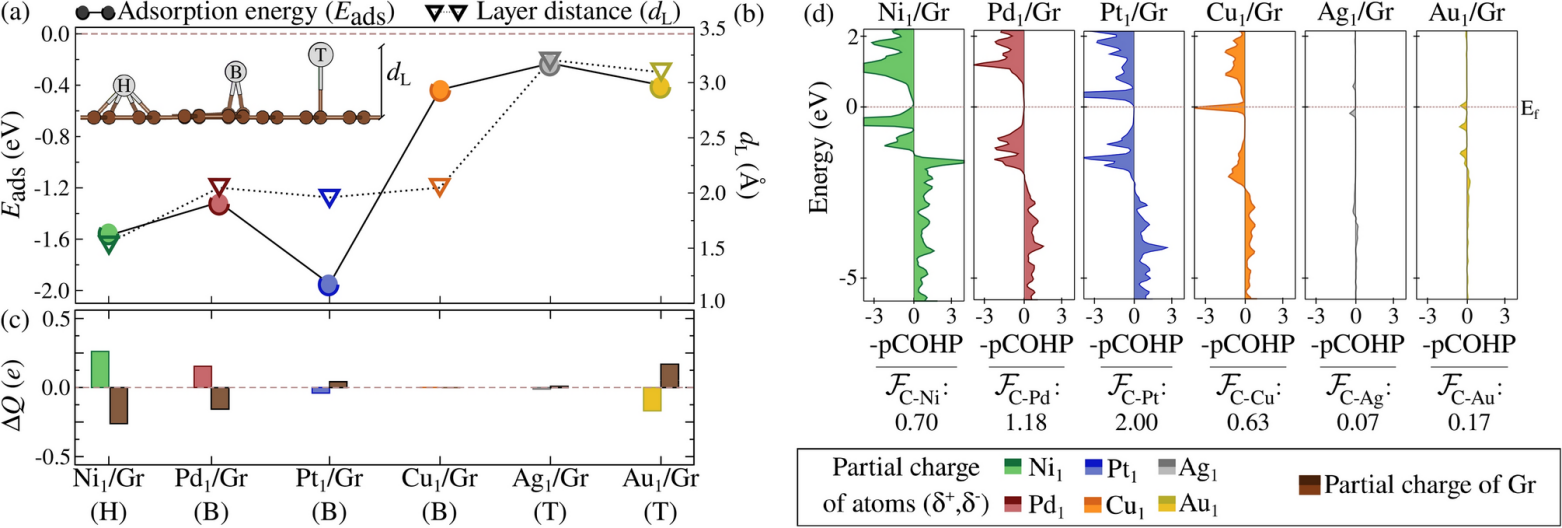
(**a**) Adsorption energy ($E_{\text {ads}}$) and (**b**) layer distance ($d_{\text {L}}$), (**c**) charge population analysis using the DDEC6 method, and (**d**) COHP and covalent bond strength ($|\mathcal {F}|$), normalized by the number of bonds, for M-C interactions. The analysis is performed for single atoms of Ni1 (green), ${\text{Pd}_1}$ (red), ${\text{Pt}_1}$ (blue), ${\text{Ag}_{1}}$ (silver), and ${\text{Au}_{1}}$ (gold), adsorbed on their most stable sites: top (T), bridge (B), and hollow (H).

### Single-atom adsorption on graphene

The most energetically stable sites for isolated metal atoms (adatoms), ${\text {M}_{1}\text {/Gr}}$ and ${\text {Au}_{1}\text {/Gr}}$, are shown in Fig. 1, alongside their respective adsorption energies ($E_{\text {ads}}$) and distances to the Gr surface ($\text {d}_{\text {L}}$). We observe a preference for ${\text {Ni}_{1}\text {/Gr}}$ at the hollow (H) site, while ${\text {Pt}_{1}\text {/Gr}}$, ${\text {Pd}_{1}\text {/Gr}}$, and ${\text {Cu}_{1}\text {/Gr}}$ prefer the bridge (B) site. Conversely, ${\text {Ag}_{1}\text {/Gr}}$ and ${\text {Au}_{1}\text {/Gr}}$ favor the top (T) site. These preferences seem to be related to the atomic radius of the transition metal and the size of the adsorption site on graphene. The hexagonal topology of Gr provides a distance of 2.85Å across the H site, while the C-C bond length ($d_{\text {C-C}}$) of 1.43Å accommodates adsorption at the B site. The relatively small atomic radius of Ni (1.25Å)^60^—among the studied metals – allows adsorption at the H site, while Pd (1.38Å), Pt (1.39Å), and Cu (1.28Å fit more comfortably between C-C bonds. The larger radii of Ag and Au (1.45Å and 1.44Å, respectively) favor adsorption on the T site. This trend suggests a progression in site preference from H (Ni) to B (Pd, Pt, Cu) to T site (Ag, Au) for elements from Groups 10 and 11, correlating with their increasing atomic radii.

The $E_{\text {ads}}$ values for the adatoms—shown in Fig. 1a—are consistent with previous studies^61,62^. Our findings further indicate a correlation between the degree of interaction ($E_{\text {ads}}$) and the distance between the adatom and graphene ($d_\text {L}$). The adsorption strength, especially in SACs, may be crucial in their catalytic performance. Group 10 elements (Ni, Pd, Pt) show strong adsorption with $E_{\text {ads}}$ values below -1.00eV, while Group 11 elements (Cu, Ag, Au) exhibit weaker interactions, with $E_{\text {ads}}$ values ranging from $-0.20$ to 0.40 eV. Figure 1b demonstrates that $d_\text {L}$ is more closely related to the adsorption site rather than $E_{\text {ads}}$, with distances of $\sim$1.55Å, $\sim$2.00Å, and $\sim$3.15 Å for the H, B, and T sites, respectively.

In addition, the adsorbed adatoms exhibit coordination numbers of 6 (on the H site), 2 (on the B site), and 1 (on the T site). A variation of $\sim$1.0Å in $d_\text {L}$ is observed for ${\text {Pt}_{1}\text {/Gr}}$ compared to (${\text {Cu}_1},{\text {Ag}_1},{\text {Au}_1}$)/Gr, which is consistent with the sharp decrease in $E_{\text {ads}}$ values.

Continuing with the electronic characterization of SACs/Gr, the partial charge population ($\Delta Q$) for the adatoms, as determined via the Density Derived Electrostatic and Chemical (DDEC6)^63^ method, is depicted in Fig. 1c. For the Group 10 metals, the charge transfer to Gr decreases along the group, ${\text {Ni}_{1}\text {/Gr}}$ donates $+$0.25*e*, which decreases for ${\text {Pd}_{1}\text {/Gr}}$ and eventually results in an inversion of ionic character for ${\text {Pt}_{1}\text {/Gr}}$, which accepts a small amount of charge. In Group 11, there is negligible charge transfer between Gr and ${\text {Cu}_{1}\text {/Gr}}$ or ${\text {Ag}_{1}\text {/Gr}}$, whereas ${\text {Au}_{1}\text {/Gr}}$ exhibits a stronger anionic character, withdrawing approximately −0.25*e* from Gr.

Therefore, it becomes clear that correlations based solely on chemical composition are insufficient for understanding the charge transfer process. The adsorption site configuration also plays a crucial role in the electrostatic contribution of SACs on Gr. For instance, by comparing Pauling electronegativity values ($\chi _\text {Ni} = 1.91$, $\chi _\text {Pd} = 2.20$, $\chi _\text {Pt} = 2.28$, $\chi _\text {Cu} = 1.90$, $\chi _\text {Ag} = 1.93$, $\chi _\text {Au} = 2.54$, $\chi _\text {C} = 2.55$)^64^, one realizes that the role of electrostatic interactions is not predictive for $E_{\text {ads}}$. Typically, charge transfer is rationalized through the difference in electronegativity ($\Delta \chi _\text {M-Gr} = \chi _\text {M} - \chi _\text {C}$) between the SACs and C, which yields differences of $\Delta \chi _\text {M-Gr} = -0.64, -0.35, -0.27, -0.65, -0.62$, and $-0.01$ for Ni, Pd, Pt, Cu, Ag, and Au, respectively.

In the case of SACs adsorbed on the B site, the trends for Pd and Pt are consistent with their respective electronegativities, where the reduction in charge donation follows $+$0.20*e*$\rightarrow$-0.05*e* as electronegativity increases ($\chi _\text {Pd} = 2.20 \rightarrow \chi _\text {Pt} = 2.28$). However, despite Cu’s lower electronegativity (1.90), it does not donate charge, likely due to its weaker interaction with Gr compared to the $E_{\text {ads}}$ values of ${\text {Pd}_{1}\text {/Gr}}$ and ${\text {Pt}_{1}\text {/Gr}}$. This can be explained by the charge-mass relationship for Cu, given its semi-filled 3d$^{10}$4s$^{1}$ valence shell. A similar behavior is observed for the T site, where the -0.20eV difference in $E_{\text {ads}}$ between ${\text {Ag}_{1}\text {/Gr}}$ and ${\text {Au}_{1}\text {/Gr}}$ results in no charge donation from Ag to Gr, due to its 4d$^{10}$5s$^{1}$ configuration. On the other hand, Au withdraws -0.25*e* from the Gr surface, highlighting the critical role of electron shielding due to its 4f$^{14}$5d$^{10}$6s$^{1}$ configuration, which stabilizes the SAC on the T site and explains its anionic character.

To assess the covalent contribution in SACs/Gr, the projected Crystal Orbital Hamilton Population (COHP)^65^ analysis, shown in Fig. 1d (denoted by -pCOHP as the M-C interaction), was conducted to estimate the covalent bond strength ($|\mathcal {F}_{\text{C-M}}|$) by integrating the COHP (ICOHP). As expected, $|\mathcal {F}_{\text{C-M}}|$ correlates with $E_{\text {ads}}$ for the adsorption of (Ni, Pd, Pt, Cu, Ag, Au)/Gr, emphasizing a stronger covalent contribution from Group 10 compared to Group 11. This supports the hypothesis of the semi-filled valence effect in Cu, Ag, and Au SACs.

Thus, when identical adsorption sites are considered, the bond strength follows the order $|\mathcal {F}_{\text{C-Pt}}|> |\mathcal {F}_{\text{C-Pd}}| > |\mathcal {F}_{\text{C-Cu}}|$ for the B site, and $|\mathcal {F}_{\text{C-Au}}| > |\mathcal {F}_{\text{C-Ag}}|$ for the T site, consistent with the behavior observed in $E_{\text {ads}}$. This suggests that $E_{\text {ads}}$ and $d_\text {L}$ for SACs/Gr systems are determined by several factors, including metal size, valence configuration, electronegativity, adsorption site, and the nature of the covalent-ionic M-C interaction. For instance, even though C and Au have similar electronegativities in $\text {Au}_{1}$/Gr, adsorption is dominated by ionic interactions, where the missing connections in T site adsorption and shielding effects lead to increased electron density on Au. These insights are critical for designing surface SACs, especially as validation data for machine learning-based models.

**Figure 2:**
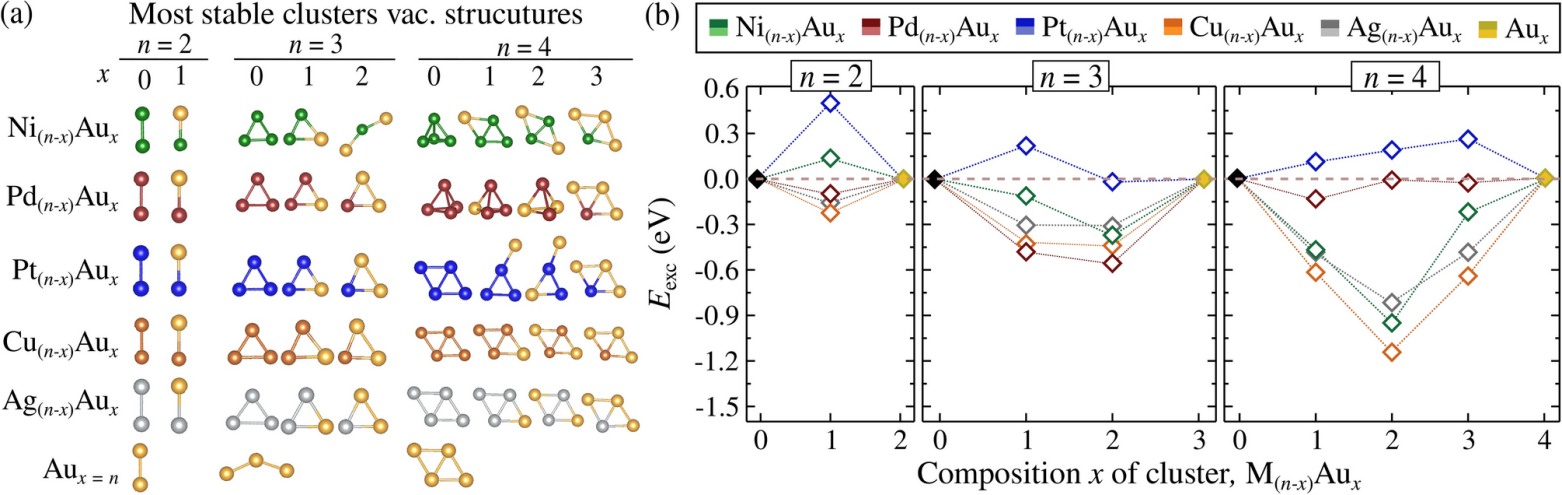
(**a**) Lowest energy structures of unary ${\text{M}_n}$ and ${\text{Au}_n}$ clusters, as well as alloy clusters (Ni, Pd, Pt, Cu, Ag)${_{(n-x)}\text{Au}_x}$, in vacuum condition (vac.) for all atomicities (*n*) and compositions (*x*). (**b**) Excess energy ($E_{\text {exc}}$) of the alloy clusters as a function of *x*, relative to the energies of the corresponding unary systems.

### Non-supported alloy clusters

To advance the understanding of mixed metal catalysts, we first conducted a detailed investigation of unary and binary (alloyed) clusters in the gas phase, vacuum, (vac.) to assess later how the Gr support influences the structure, stability, and electronic properties of these systems. The energetically lowest configurations for unary and binary clusters in the gas phase (vac.) are presented in Fig. 2a. Our findings for unary systems align well with previous theoretical and experimental studies^61,66,67^, as depicted in Table S1 (Supporting Information), where binding energies are compared. Additionally, all high-energy isomers and their relative energies ($E_{\text {rel}}$), along with detailed structural properties, are provided in Fig. S1–S3, as well as in the Supporting Information.

To assess the stability of the alloys, we calculated the excess energy ($E_{\text {exc}}$, using Eq. 2) relative to unary ${\text {Au}_n}$ and ${\text {M}_{n}}$ clusters. This provided a reference for the stability of the alloys compared to chemical segregation. The results for the non-supported systems are shown in Figure 2(b). We found that (Ni, Cu, Ag)${_{(n-x)}}{\text {Au}_x}$ alloys exhibited favorable formation, achieving maximum stability at a 1:1 metal-to-Au ratio. Specifically, the highest stabilities were observed for $n = 4$, with $E_{\text {exc}}$ values of $-0.92$ eV, $-1.13$ eV, and $-0.83$ eV for ${\text {Ni}_{2}\text {Au}_{2}}$, ${\text {Cu}_{2}\text {Au}_{2}}$, and ${\text {Ag}_{2}\text {Au}_{2}}$, respectively. In contrast, (Pd, Pt)${_{(n-x)}\text {Au}_{x}}$ alloys displayed weaker affinity with Au, particularly for compositions like $x = 3$ and 4 for Pd and all compositions for Pt. For example, the PtAu tetramers showed decreasing stability as the number of Au atoms increased, reaching +0.26eV for ${\text {Pt}_{1}\text {Au}_{3}}$. Given that the thermal energy (*kT*) at room temperature ($\sim {0.03} \text{eV}$) is small, some alloys are near the threshold of stability and segregation in the gas phase, such as ${\text {Pd}_{2}\text {Au}_{2}}$ and ${\text {Pt}_{1}\text {Au}_{2}}$, indicating the potential impact of a 2D support on their catalytic behavior.

Before examining supported alloys, we further explored the ionic-covalent interaction mechanisms (e.g., charge transfer and sharing process) between metal atoms. This involved a comparative study using DDEC6 and Bader methodologies (Figs. S4–S6 in Supporting Information) to evaluate ionic contributions using COHP analysis to rationalize covalent interactions. While the Bader method is widely used, it is sometimes criticized for not accurately reproducing electrostatic potentials and dipole moments, as it can yield non-linear attractors, leading to undefined charges^68^. In contrast, DDEC6 agreed well with chemical insights based on Pauling electronegativities^64^.

**Figure 3:**
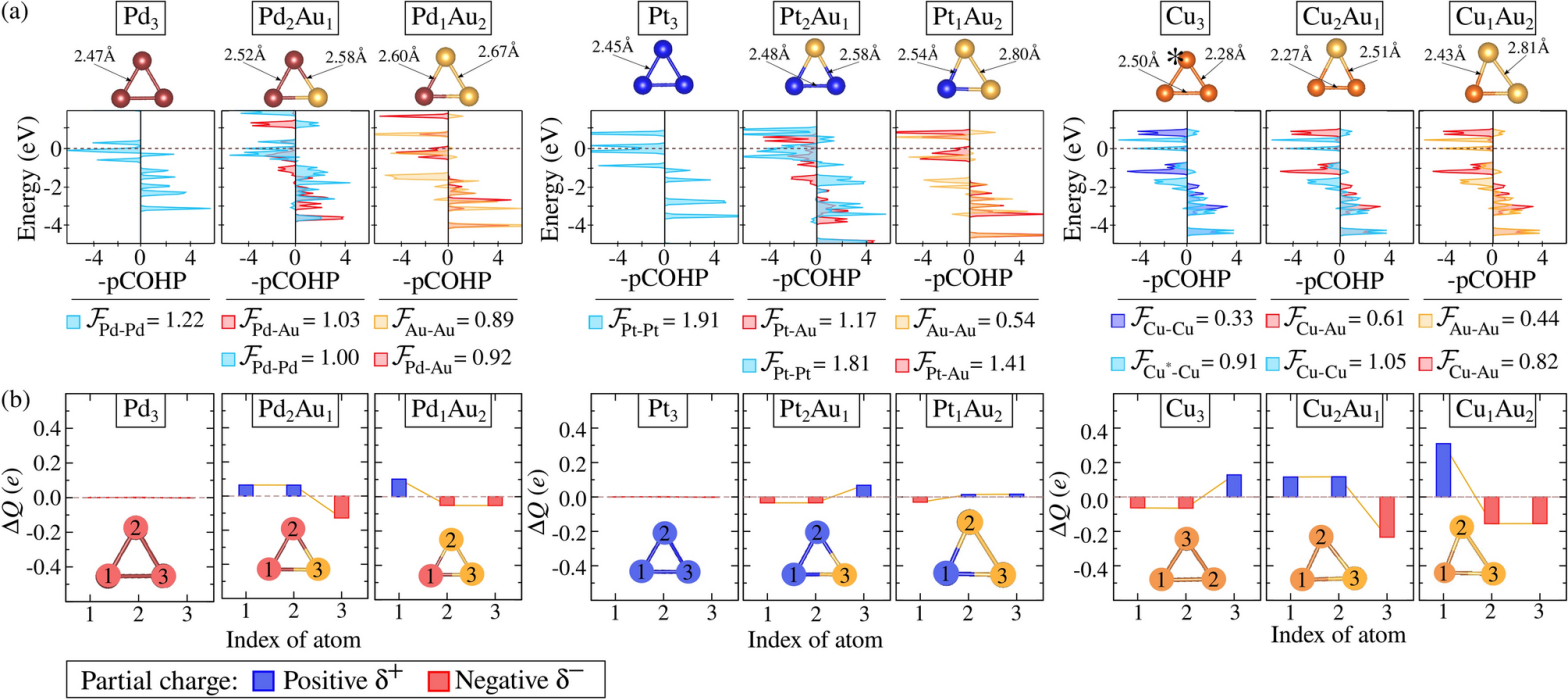
(**a**) COHP analysis for unary and binary trimer clusters in the gas phase, focusing on M–M and M–Au interactions. The covalent bond strength, $|\mathcal {F}_{\text {M-Au, Au-Au, M-M}}|$, is determined through the integration of COHP (ICOHP) values, provided in eV and normalized by the number of equivalent bonds. The asterisk (*) indicates configurations where a given element adopts a non-similar structural arrangement. (**b**) Partial charge population ($\Delta Q$) for each indexed atom in unary and binary trimer clusters in the gas phase, obtained using the DDEC6 methodology. Blue and red bar charts represent negative ($\delta ^-$) and positive charges ($\delta ^+$).

The projected COHP (pCOHP) analysis, shown in Fig. 3a, helped us determine the covalent bond strength ($|\mathcal {F}_{\text {M-Au, Au-Au,}}$ $_\text {M-M}|$), derived from the integration of COHP (ICOHP) values. The stability of (Pd, Pt, Cu)${_{(3-x)}\text{Au}_{x}}$ mixtures, as indicated by $E_{\text {exc}}$, was correlated with interactions between different metal atoms. The tendency towards alloy formation (or segregation) was rationalized by comparing the M-Au and M-M interactions in the alloys. Full COHP analysis for unary and binary clusters in the gas phase can be found in Figure S7-S11. We found that alloy formation is primarily driven by a balance between bonding forces, where covalent interactions between chemically distinct atoms are stronger than those between similar atoms. Specifically, $|\mathcal {F}_{\text {M-Au}}| > |\mathcal {F}_{\text {M-M,Au-Au}}|$, which favors alloy formation. For example, $|\mathcal {F}_{\text {Pd-Au}}| > |\mathcal {F}_{\text {Pd-Pd, Au-Au}}|$ in ${\text {Pd}_{2}\text {Au}_{1}}$ and ${\text {Pd}_{1}\text {Au}_{2}}$, i.e., Pd-Au bonds were stronger by 0.03eV, promoting alloying, whereas ${\text {Pt}_{2}\text {Au}_{1}}$ showed segregation ($|\mathcal {F}_{\text {Pt-Pt}}| > |\mathcal {F}_{\text {Pt-Au}}|$) due to stronger Pt-Pt bonds (0.64eV). However, ${\text {Pt}_{1}\text {Au}_{2}}$ formed a stable mixture as the Pt-Au bond strength surpassed that of Au-Au by 0.87eV ($|\mathcal {F}_{\text {Pt-Au}}| > |\mathcal {F}_{\text {Au-Au}}|$).

Since $|\mathcal {F}_{\text {M-Au,Au-Au,M-M}}|$ only represents the covalent portion of the stability energy, we also considered electrostatic contributions, which play a significant role in systems like ${\text {Cu}_{(n-x)}\text {Au}_{x}}$. For instance, in ${\text {Cu}_{2}\text {Au}_{1}}$, the bond strength $|\mathcal {F}_{\text {Au-Au}}| > |\mathcal {F}_{\text {Cu-Au}}|$. Charge population mapping using DDEC6 (Fig. 3b and Figs. S4–S6 in Supporting Information) revealed that electrostatic interactions significantly influence thermodynamic stability. The polarization of the ${\text {Cu}^{\delta +}-\text {Au}^{\delta -}}$ species, due to charge transfer from Cu to Au in $\text {Cu}_{2}\text {Au}_{1}$, supports alloy formation despite weaker covalent interactions. In contrast, ${\text {Pd}_{(3-x)}\text {Au}_{x}}$ and ${\text {Pt}_{(3-x)}\text {Au}_{x}}$ exhibited different behaviors, with $\text {Pd}^{\delta +}-\text {Au}^{\delta -}$ following Pauling electronegativity trends, while Pt and Au in PtAu alloys competed for charge. This competition led to insufficient stabilization through covalent bonds alone, emphasizing the importance of both covalent and electrostatic contributions in M-Au alloy stabilization.

### Adsorbed clusters on graphene

**Figure 4:**
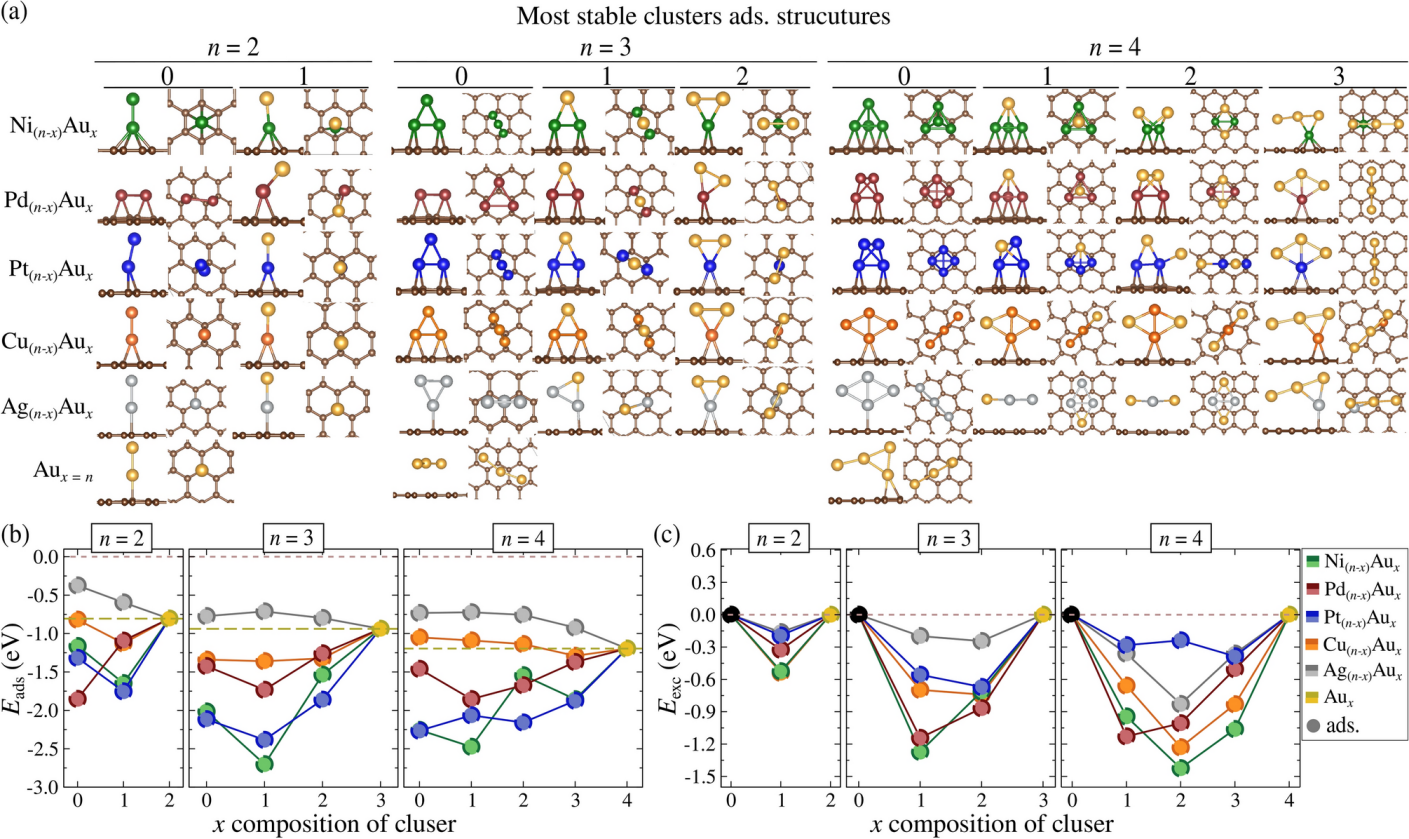
(**a**) Lowest energy structures of clusters adsorbed on graphene (ads.) for unary ($x=0,x=n$) and binary ($0<x<n$) configurations, across all compositions (*x*). (**b**) Adsorption energy ($E_{\text {ads}}$) and (**c**) excess energy ($E_{\text {exc}}$) in eV for dimers ($n=2$), trimers ($n=3$), and tetramers ($n=4$) in the adsorbed state. The colors green, red, blue, orange, and silver represent unary and alloy clusters of transition metals: M = Ni, Pd, Pt, Cu, and Ag, respectively.

The impact of Gr adsorption on the structural, energetic, and catalytic properties of unary and binary clusters was explored, building on our prior gas-phase investigation. Figure 4a shows the energetically lowest configurations of unary and binary clusters adsorbed on Gr (ads.). The stability of these clusters is quantified by the adsorption energy ($E_{\text {ads}}$, Eq. 1), while the favorability of alloy mixing is evaluated through $E_{\text {exc}}$, influenced by surface interactions. Figure 4b presents $E_{\text {ads}}$ for unary and binary clusters. Our calculated $E_{\text {ads}}$ values for unary ${\text {M}_{n}\text {/Gr}}$ and ${\text {Au}_{x}\text {/Gr}}$ are consistent with previous theoretical studies^61,62,66,67,69^ (see Table S2 to S5 in the Supporting Information for all isomers).

For dimers ($n = 2$), the $\text {M}_{1}\text {Au}_{1}$ clusters generally exhibit stronger adsorption on Gr than their unary M$_{2}$ counterparts, with Pd$_{2}$ being an exception due to its strong adsorption at bridge sites on Gr. Notably, despite a weaker Pd$_{2}$ connection, the binary Pd$_{1}$Au$_{1}$ system exhibits a higher $|E_{\text {ads}}|$ by approximately 0.17eV, illustrating the effect of alloy composition on adsorption. The substitution of M by Au consistently improves $|E_{\text {ads}}|$, as observed across all atomicities (see Table S6–S8 in the Supporting Information). This suggests that alloy formation with Au enhances adsorption on Gr, especially when M serves as an anchoring element for Au-based clusters. For example, Ni$_{1}$Au$_{1}$ and Pt$_{1}$Au$_{1}$ show the highest $|E_{\text {ads}}|$ values, around 0.95eV, with Ni playing an anchoring role. Conversely, Ag$_{1}$Au$_{1}$ demonstrates unfavorable adsorption compared to unary clusters due to the larger atomic radii of both Ag and Au, leading to a reduction in adsorption energy by 0.22 eV.

For trimers ($n = 3$), clusters like Ni$_{3}$, Pt$_{3}$, and Cu$_{3}$ anchor through two contact points on Gr, while Pd$_{3}$ follows the dimer behavior, anchoring at three bridge sites on neighboring hexagons. Alloy trimers highlight Au’s lack of affinity for Gr, as Au atoms typically occupy non-bonded positions. When the composition shifts from M$_{2}$Au$_{1}$ to M$_{1}$Au$_{2}$, the number of contact points decreases, reducing adsorption stability (e.g., $E_{\text {ads}}$ for Ni$_{2}$Au$_{1}$ $\rightarrow$ Ni$_{1}$Au$_{2}$ decreases from -1.86 to -1.54eV). This trend is consistent for all alloys, including Pd$_{2}$Au$_{1}$ $\rightarrow$ Pd$_{1}$Au$_{2}$ (-1.73eV $\rightarrow$ -1.26eV), Pt$_{2}$Au$_{1}$ $\rightarrow$ Pt$_{1}$Au$_{2}$ (-2.38eV $\rightarrow$ -1.86eV), and Cu$_{2}$Au$_{1}$ $\rightarrow$ Cu$_{1}$Au$_{2}$ (-1.36 eV $\rightarrow$ -1.32eV), reinforcing the role of M atoms as the primary anchoring points. Ag$_{(3-x)}$Au$_{x}$ alloys, as in the dimer case, exhibit weaker adsorption than the unary Au$_{3}$ due to the larger atomic radii of Ag and Au, preventing effective anchoring.

For tetramers ($n = 4$), we found that only the Au-based alloys in Group 10 (i.e., Ni, Pd, and Pt) exhibit significant morphological changes when transitioning from the gas phase to the adsorbed state. This transition results in more efficient adsorption, as more M atoms bind to the Gr surface. For example, the Ni$_{3}$Au cluster transitions from a planar configuration in the gas phase to a tetrahedral structure upon adsorption, where three Ni atoms act as contact points with the surface. The trend observed in $n = 3$ clusters, where replacing M with Au reduces the number of contact points on Gr and decreases the adsorption energy, is also observed here.

Taking ${\text {Pd}_{(4-x)}\text {Au}_{x}}$ as a representative system, we observe a clear evolution in the number of contact points as the Au content increases ($x = 1 \rightarrow 2 \rightarrow 3$). The number of contact points decreases from 3 to 2 to 1 as the Pd atoms remain consistently bonded to Gr, while the Au atoms remain exposed to the environment. This reduction in contact points correlates with a weakening of the interaction between the cluster and the graphene surface, as evidenced by a decrease in $E_{\text {ads}}$ from -1.85eV to -1.67eV to -1.37eV for the $\text {Pd}_{1}\text {Au}_{3}$, $\text {Pd}_{2}\text {Au}_{2}$, and $\text {Pd}_{3}\text {Au}_{1}$ clusters, respectively.

For Au-based alloys from Group 11, no significant morphological changes were observed between the gas-phase and adsorbed states for both $\text {Cu}_{(4-x)}\text {Au}_{x}$ and $\text {Ag}_{(4-x)}\text {Au}_{x}$ clusters. These systems tend to maintain planar configurations, and when combined with Au, these semi-filled orbitals result in weak binding to the graphene surface. These clusters exhibit a lack of adsorption affinity, which we attribute to the semi-filled valence shells of Cu (3d$^{10}$4s$^{1}$) and Ag (4d$^{10}$5s$^{1}$). Combined with Au, these semi-filled orbitals result in weak binding to the graphene surface, characterized by its delocalized $p_z$ electrons. Our results suggest that metallic species with high electron density in their valence shells when combined with Au, form loosely bound systems on high-electron-density surfaces like Gr.

The preceding discussion highlights the superior adsorption behavior of Group 10 alloys (Ni, Pd, Pt)$_{(n-x)}\text {Au}_{x}$ in comparison to Group 11 alloys (Cu, Ag)$_{(n-x)}\text {Au}_{x}$, which can be attributed to the unfilled valence shells of Group 10 metals and their impact on $E_{\text {ads}}$. This suggests that Group 10 metals are more effective stabilizing agents for Au-based nanostructures on Gr, with the difference in electronic configuration between these two groups playing a key role. Specifically, Group 10 metals exhibit flexibility in their *d*-state configurations ($nd^8$, $nd^9$, $nd^{10}$). In contrast, the filled $nd^{10}$ shell and additional $ns^{1}$ electron of noble metals in Group 11 limit the number of simultaneous bonds that can be formed. This flexibility is crucial for a stabilizing agent, as it must be capable of binding both to the substrate (in this case, graphene) and to other metal atoms (in the alloy), thereby forming a bridge-like structure that can effectively anchor Au atoms onto the Gr surface.

For the thermodynamic stability of alloys under adsorbed conditions, as shown in Fig. 4c, an additional consideration in our approach involves the potential changes in morphological configurations across different *x* compositions, specifically in terms of altering the number of contact points. It is crucial to account for the possible influence of $E_{\text {ads}}$ on the $E_{\text {exc}}$ values, as it may arise from variations in the contact points in the unary $\text {M}_{n}$ and $\text {Au}_{n}$ reference systems. To better understand the effect of adsorption on $E_{\text {exc}}$ and to minimize any undesirable interference, we have considered the total energy of adsorbed unary $\text {M}_{n}\text {/Gr}$ and $\text {Au}_{x}\text {/Gr}$ clusters in a meta-stable configuration. This configuration maintains a similar morphology (i.e., preserving a comparable number of contact points) to the respective $\text{M}_{(\textit{n}-\textit{x})}\text{Au}_{\textit{x}}$ /Gr alloy.

Adsorption on Gr leads to favorable alloy formation for all values of atomicity *n* and composition *x*. The highest stability for (Ni, Cu, Ag)$_{(n-x)} \text {Au}_{x}\text {/Gr}$ clusters was found to occur at a 1 : 1 ratio in the adsorbed phase. Notably, $\text {Ni}_{2}\text {Au}_{2}$ exhibits a significant increase in stability, becoming -0.51eV more stable upon transitioning from the gas phase to adsorption. In contrast, CuAu and AgAu follow the trend observed in non-adsorbed systems, favoring alloy formation. Additionally, similar to NiAu, the adsorption of (Pd, Pt)$_{(n-x)}\text {Au}_{x}$ alloys shows a marked enhancement in the chemical affinity of Pd and Pt for alloying with Au. As a result, the PdAu system transitions from being on the verge of segregation to achieving high stability, as indicated by the $E_{\text {exc}}$ range. The presence of the Gr surface effectively prevents segregation in PdAu and PtAu alloys. For example, $\text {Pd}_{2}\text {Au}_{2}$ stabilizes significantly, with $E_{\text {exc}}$ shifting from -3meV to -1.01eV, while $\text {Pt}_{1}\text {Au}_{3}$, previously the least stable $n = 4$ configuration, is stabilized by -0.65eV.

Our findings regarding thermodynamic stability align with the observed adsorption stability trends, where Au-alloys with greater $E_{\text {ads}}$ (particularly those involving Group 10 metals) exhibit enhanced alloy mixing. This indicates a significant surface-mediated influence on the favorability of their formation. The surface effects manifest in two key ways: first, by augmenting the stability of alloys that are already thermodynamically stable, and second, by facilitating the formation of alloys that were otherwise unfavorable in the gas phase, thus preventing segregation on the Gr surface.

**Figure 5:**
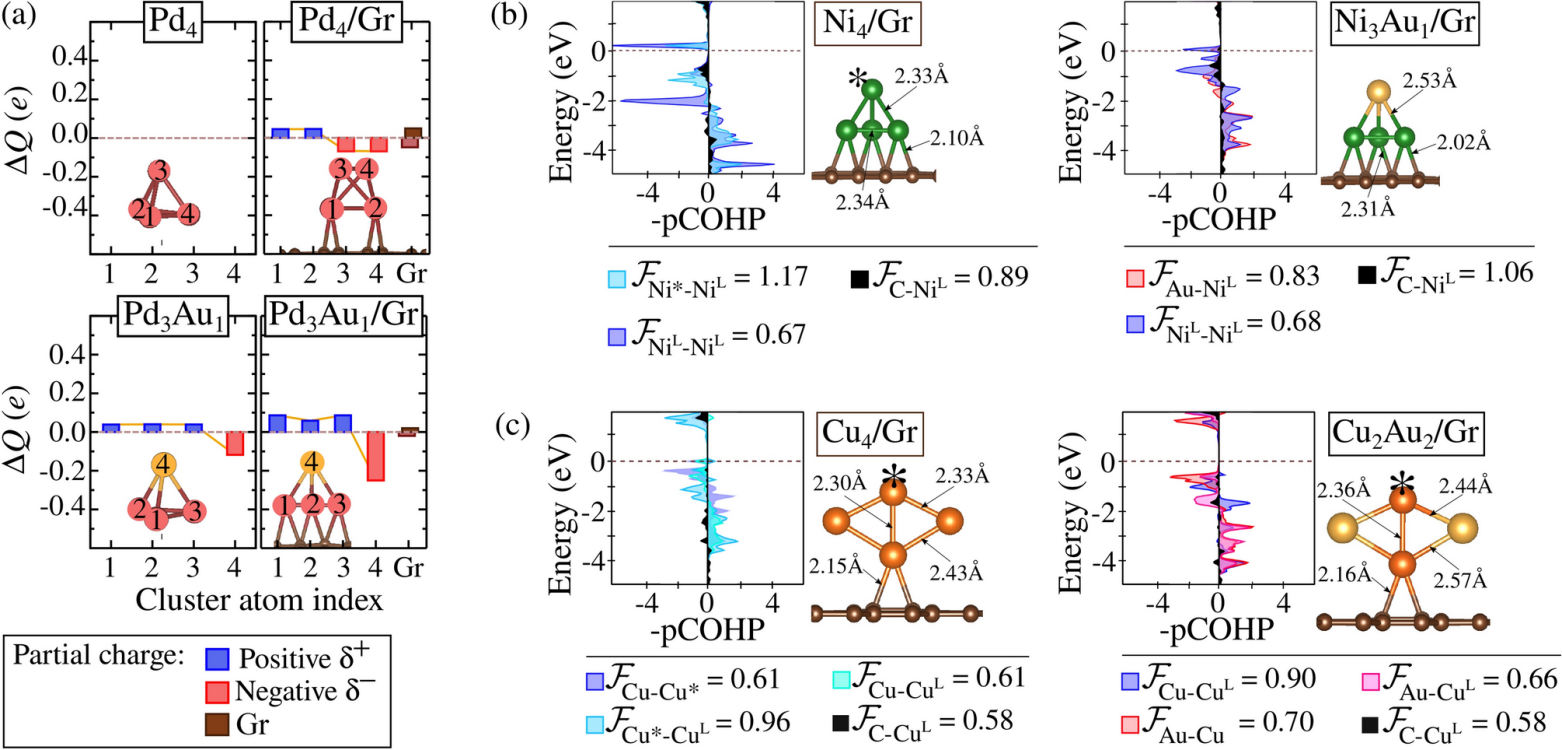
(**a**) DDEC6 charge population for each indexed atom in $\text {Pd}_4$ and $\text {Pd}_{3}\text {Au}_{1}$ clusters in both the gas phase and adsorbed on graphene. (**b**) COHP analysis for M-M, M-Au, and C-M interactions in $\text {Ni}_{4}\text {/Gr}$ and $\text {Ni}_{3}\text {Au}_{1}\text {/Gr}$. (**c**) COHP analysis for M-M, M-Au, and C-M interactions in $\text {Cu}_{4}\text {/Gr}$ and $\text {Cu}_{2}\text {Au}_{2}\text {/Gr}$. The covalent bond strength, $|\mathcal {F}_{\text {M-Au,Au-Au,M-M,C-M}}|$, is derived from the integration of COHP (ICOHP) values, presented in eV and normalized by the number of equivalent bonds. The $^\text {L}$ notation denotes the metal atoms bonded to Gr, while the asterisk (*) indicates configurations where a given element adopts a non-similar structural arrangement.

### Electronic properties and catalytic potential

The analytical framework for understanding ionic-covalent interactions in non-supported systems was also applied to the $\text{M}_{(\textit{n}-\textit{x})}\text{Au}_{\textit{x}}$ /Gr systems. Here, metal–metal and metal–Gr bonds were evaluated using DDEC6 and Bader charge analyses for electrostatic contributions (Figs. S4–S6), along with covalent bond strength calculations ($|\mathcal {F}_{\text {M-Au,Au-Au,M-M,C-M}}|$) derived from the integration of COHP (Figs. S12–S16).

Figure 5a shows the general impact of surface adsorption on charge distribution, promoting partial ionic sites in unary clusters. For example, in $\text {Pd}_4$, the atoms directly bonded to Gr ($\text {Pd}^{\text{L}}$) acquire a cationic character ($+$0.05*e*), while the exposed atoms become anionic (-0.07*e*). Additionally, Au-alloys adsorb on Gr through cationic M sites (${\text {M}^{\delta +}}$), intensifying the polarization of ${\text {M}^{\text{L}\delta +}-\text {Au}^{\delta -}}$ bonds (in which $^\text {L}$ refers to the metal bonded to the Gr). This enhances electrostatic contributions, with Au in $\text {Pd}_{3}\text {Au}_{1}$ being charged by -0.15*e* upon transitioning from the gas phase to the adsorbed state. Notably, $\text {M}^\text {L}$ atoms exhibit two behaviors: slightly anionic (${\text {M}^{\text{L}\delta -}}$, as in $\text {Ni}_2$, $\text {Pt}_{2}$, $\text {Au}_{2}$, $\text {Au}_{2}$, $\text {Pt}_{4}$, $\text {Pt}_{3}\text {Au}_{1}$, $\text {Pt}_{2}\text {Au}_{2}$, $\text {Pt}_{1}\text {Au}_{3}$) or cationic ($\text {M}^{\text{L}\delta +}$ for others), with the former more prominent in unary systems.

The enhanced adsorption stability of alloys over unary clusters can be attributed to the increase in $|\mathcal {F}_{\text {C-M}^\text {L}}|$ upon Au incorporation. Figure 5b show this behaviour for $\text {Ni}_{4}$, where substituting an exposed Ni with Au strengthens the $|\mathcal {F}_{\text {C-Ni}^\text {L}}|$ in 0.17eV. A similar effect is observed in $\text {Cu}_{2}\text {Au}_{2}\text {/Gr}$ (Fig. 5c), where Au remains fully exposed, and $|\mathcal {F}_{\text {C-Cu}^\text {L}}|$ remains comparable to $\text {Cu}_{4}\text {/Gr}$, with a $E_{\text {ads}}$ difference of -0.09eV, linked to electrostatic contributions as $\text {Cu}^{L\delta +}$ becomes more cationic by $+$0.10*e* (Fig. S5).

**Figure 6:**
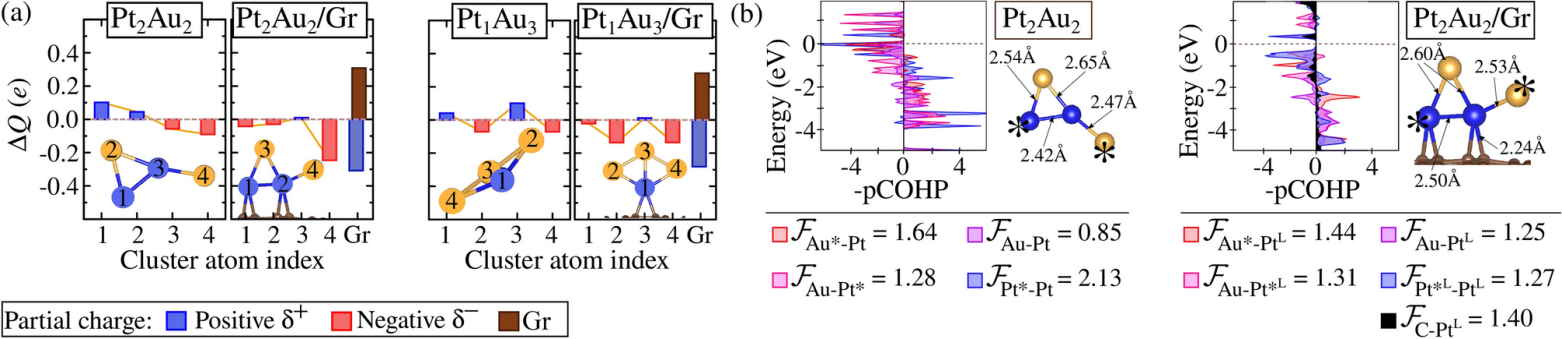
(**a**) DDEC6 charge population for each indexed atom in the cluster $\text {Pt}_{2}\text {Au}_{2}$ and $\text {Pt}_{1}\text {Au}_{3}$ in both the gas phase and adsorbed on graphene. (**b**) COHP analysis for $\text {Pt}_{2}\text {Au}_{2}$ in the gas phase and in the adsorbed state, where the covalent bond strength, $|\mathcal {F}_{\text {Pt-Au,Au-Au,Pt-Pt,C-Pt}}|$, is obtained from the integration of COHP (ICOHP) values, expressed in eV and normalized by the number of equivalent bonds. The $^\text {L}$ notation indicates the metal atoms that are bonded to Gr, while the asterisk (*) denotes configurations where a given element exhibits a non-similar structural arrangement.

The thermodynamic stabilization of alloy formation upon adsorption is thus linked to increased electrostatic interactions between metals in the cluster induced by adsorption. However, for PtAu alloys, the competition between Pt and Au for charge induces a different stabilization mechanism on Gr. Adsorption increases the anionic character of all sites, as in $\text {Pt}_{2}\text {Au}_2$, which withdraws -0.30*e* from the surface, forming $\text {Pt}_{2}\text {Au}{2}^{\delta -}\text {/Gr}$ (Fig. 6a). The covalent weakening of $|\mathcal {F}_{\text {Pt*-Pt}}|$ by 0.86 eV upon adsorption, as shown in Fig. 6b, favors alloying by limiting the segregation tendency observed in the gas phase. This weakening of $|\mathcal {F}_{\text {M}^\text {L}\text {-M}^\text {L}}|$ is consistent across all unary and binary clusters (Figs. S12–S16).

Thus, the stability of mixing upon adsorption is associated with the weakening of homonuclear covalent bonds (M–M and Au–Au) while M–Au interactions become dominant. Moreover, contact with Gr enhances polarization in $\text {M}^{L\delta +}\text {-Au}^{\delta -}$ bonds, increasing the electrostatic contribution to alloy formation stability.

To provide quantitative insights into the catalytic potential of unary clusters and Au-alloys, the *d*-band center ($\varepsilon _{\text {d}}$) model was employed, establishing a correlation with their chemical reactivity. Standard reaction processes, specifically HER, OER, and ORR, were used as references based on experimental syntheses and theoretical characterizations of their electronic structures^70–75^.

In this context, we propose an optimal $\varepsilon _{\text {d}}$ range of $-1.0$ to 2.0 eV, based on the catalytic efficiencies detailed in these studies, as summarized in Table S10. According to the Sabatier’s principle, an efficient catalytic process requires a balanced interaction between the catalyst and the reactants, where the binding is neither too strong nor too weak. A *d*-band center within this proposed range suggests a binding energy that facilitates reaction kinetics while avoiding excessive adsorption, which could hinder product release. As expected from a generalized perspective, this range supports sufficient interaction strength to accelerate reaction rates without impeding the overall catalytic cycle. We also provided, for a comprehensive electronic characterization, the density of states (DOS) partitioned into the $s$- and $d$-states of the M and Au metals, along with the magnetic moments of these clusters, presented in Figs. S17–S21 and Table S11.

**Figure 7:**
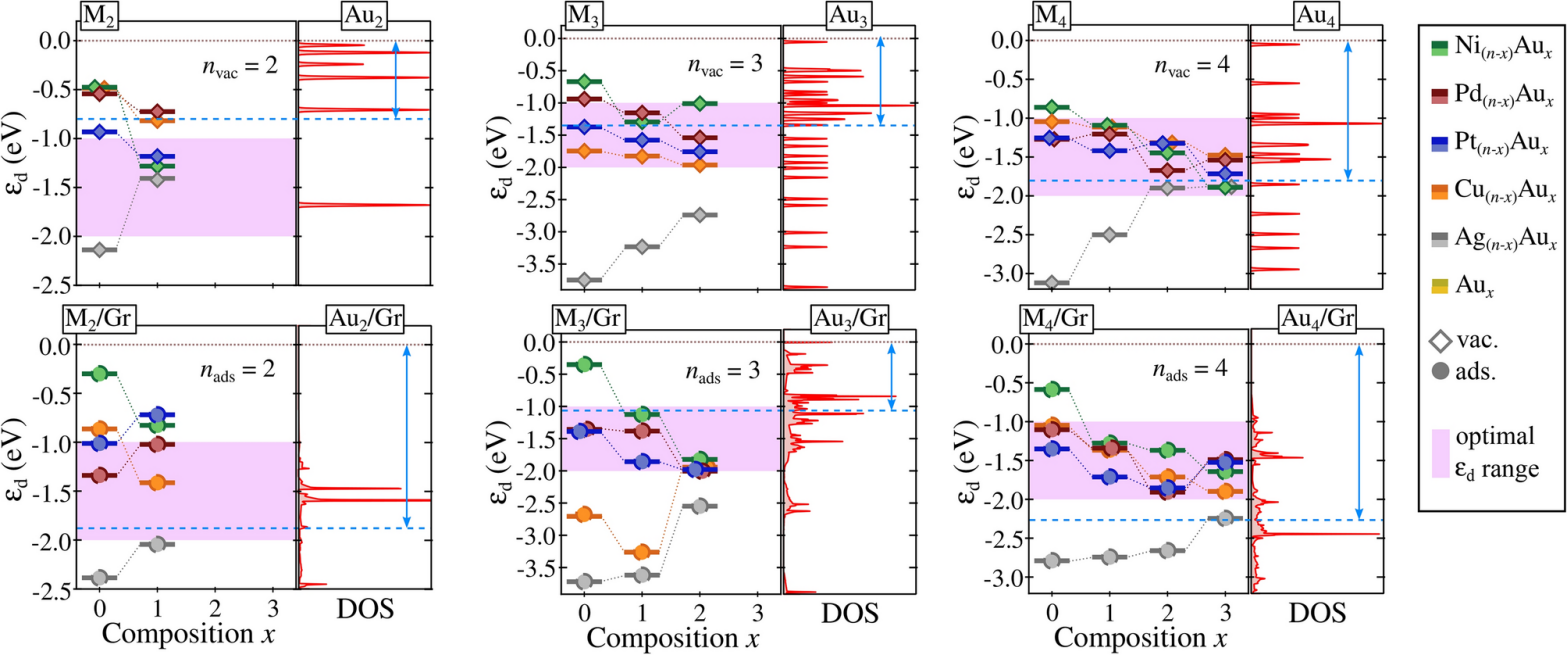
The *d*-band center ($\varepsilon _{\text {d}}$) for dimers, trimers, and tetramers of unary and alloy clusters in both the gas phase (vac.) and adsorbed on graphene (ads.). The colors green, red, blue, orange, and silver represent the compositions of the $\text{M}_{(\textit{n}-\textit{x})}\text{Au}_{\textit{x}}$ clusters, with M = Ni, Pd, Pt, Cu, and Ag, respectively. For all atomicities in both the vac. and ads. states, the density of states (DOS) for pure $\text {Au}_{n}$ clusters is also presented.

The $\varepsilon _{\text {d}}$ values for our unary and alloy clusters, both in the gas phase and adsorbed on graphene, are shown in Fig. 7, with the pink shaded region representing the optimal $\varepsilon _{\text {d}}$ range. Incorporating Au into unary $\text {M}_{n}$, systems gradually shift their $\varepsilon _{\text {d}}$ values towards this optimal range. For example, $\text {Ni}_{3}$ has an $\varepsilon _{\text {d}}$ value closer to the $\text {E}_\text {f}$ at −0.34 eV. In contrast, the inclusion of Au leads to $\varepsilon _{\text {d}}$ values of −1.11 eV and −1.80 eV for $\text {Ni}_{2}\text {Au}_{1}$ and $\text {Ni}_{1}\text {Au}_{2}$, respectively. This demonstrates that Au enhances the catalytic properties of the metal M in most systems, effectively modulating the $\varepsilon _{\text {d}}$ to approximate that of pure $\text {Au}_{n}$.

However, this modulation is not observed in PtAu alloys. In both vacuum and adsorbed conditions, this approximation occurs only for $n = 4$, while a contrasting behavior is noted for clusters with 2 and 3 atoms. A disruption in the linear relationship of Au modulation in alloys inducted by the surface indicates that the $\varepsilon _{\text {d}}$ extension may either increase or decrease, influenced by the shifting $\varepsilon _{\text {d}}$ values of the $\text {M}_{n}$ systems.

For instance, $\text {Ni}_{n}$ exhibits an $\varepsilon _{\text {d}}$ close to $\text {E}_\text {f}$ upon adsorption. In contrast, $\text {Au}_{x}$ shifts further away, increasing the difference between their $\varepsilon _{\text {d}}$ values and enhancing the modulation of the NiAu alloy by Au. Similarly, the $\varepsilon _{\text {d}}$ values for $\text {Ag}_{4}\text {/Gr}$ and $\text {Au}_{4}\text {/Gr}$ are closely aligned, thereby reducing the extent of Au modulation in AgAu alloys.

## Discussion

This study addressed the stability challenges of Au-based nanostructures, particularly in their application as heterogeneous catalysts, where substrate anchoring is essential. To tackle this, we developed and employed an automated workflow approach based on *ab initio* DFT calculations with D3 semi-empirical vdW corrections. This workflow enabled us to evaluate the adsorptive and thermodynamic stability, as well as the catalytic potential, of Au-based clusters alloyed with Group 10 (Ni, Pd, Pt) and Group 11 (Cu, Ag) metals on pristine Gr. Here, we used this workflow for raw data acquisition across the entire project; we automated submission, monitoring, and data retrieval processes, saving time and ensuring the correctness of our simulation protocols. Additionally, the workflow ensured that our work was reproducible and adhered to FAIR and TRUE data principles, enhancing the reliability and accessibility of our findings. This automated methodology provides a valuable tool for theorists and experimentalists studying similar systems. It facilitates more profound insights into the physics of complex catalytic processes while seamlessly managing computational intricacies. Our findings indicate that Group 10 alloys demonstrate promising adherence to the surface due to their enhanced adsorption energy compared to pure $\text {Au}_{n}$ clusters. This increased efficacy can be attributed to the smaller atomic radii of Group 10 metals, which allows for adsorption at sites that promote effective interactions with Gr, specifically at the C-C bond sites (B site) and within carbon hexagonal cavities (H site). Furthermore, electronegativity plays a crucial role; transition metal atoms closer to C exhibit stronger covalent contributions to the M-C bonds. The adsorption of alloys occurs via the metal M, which remains bound to Gr. In contrast, Au remains exposed, forming a partially ionic bridge-like structure $\text {Au}^{\delta -}-\text {M}^{\delta +}\text {/Gr}$ that is more polarized than the cluster in the gas phase. The M-C bonds are strengthened by Au incorporation, as evidenced by the higher $|E_{\text {ads}}|$ for $\text {M}_{(n-x)}\text {Au}_{x}$ alloys compared to their unary $\text {M}_{n}$ counterparts. A thermodynamic analysis of the mixing tendency between M and Au, assessed through excess energy, indicated that alloy formation is thermodynamically favorable on Gr. Alloys in the gas phase become even more stable upon adsorption, while those exhibiting phase segregation tendencies display thermodynamically favorable mixing. This stabilization mechanism is driven by the weakening of both covalent and electrostatic contributions in the bonds between homologous atoms, where the repulsion between $\text {M}^{\delta +}-\text {M}^{\delta +}$ and $\text {Au}^{\delta -}-\text {Au}^{\delta -}$ is counterbalanced by the covalent strengthening of the M–Au bond and the attraction between $\text {Au}^{\delta -}$ and $\text {M}^{\delta +}$. Consequently, it holds that $|\mathcal {F}_{\text {M-Au}}| > |\mathcal {F}_{\text {Au-Au, M-M}}|$. Notably, only PtAu alloys exhibit a weakening of all bonds between the cluster metals due to competition for the charge between Pt and Au, given their similar electronegativities. However, the Pt-Pt bonds are most significantly weakened because of their direct contact with Gr. The adsorption of $\text {Pt}_{(n-x)}\text {Au}_{x}$ allows the alloy to draw charge from the Gr surface, resulting in a partially charged cluster, $\text {(PtAu)}^{\delta -}\text {/Gr}$, which renders alloy formation thermodynamically favorable. In addition to stabilizing Au on Gr, Au-based alloys are essential for achieving catalytic properties comparable to those of pure Au nanostructures for applications in heterogeneous catalysis. Using the *d*-band model, we employed the *d*-band center ($\varepsilon _{\text {d}}$) to indicate the catalytic potential for unary systems and examined its variation with increasing Au incorporation. We observed that the modulation of $\varepsilon _{\text {d}}$ in alloys by Au shifts the $\varepsilon _{\text {d}}$ energy value closer to that of pure Au systems, underscoring the overlap in catalytic properties between Au and the alloys. Establishing an optimal $\varepsilon _{\text {d}}$ range, Group 10 alloys emerge as promising catalyst candidates for hydrogen evolution reaction and oxygen evolution reaction applications. Thus, our findings demonstrate that forming Au-based alloys is an effective strategy for stabilizing Au nanostructures on substrates with low affinity while maintaining comparable catalytic potential.

## Methods

We employed spin-polarized density functional theory (DFT)^76,77^ through the generalized gradient approximation as formulated by Perdew, Burke, and Ernzerhof (PBE)^78^ for the calculations of all systems. The Kohn–Sham equations were solved using the Vienna *Ab initio* Simulation Package (VASP)^79,80^, which adopts the all-electron projected augmented wave (PAW) method^81,82^ to describe the Kohn–Sham orbitals. The D3 empirical approach for van der Waals corrections, as proposed by Grimme^83^, was also used to consider long-range interactions in our systems. The following configurations were explicitly considered for the valence electrons: C ($2s^2$, $2p^2$), Ni ($3d^8$, $4s^2$), Pd ($4d^9$, $5s^1$), Pt ($5d^9$, $6s^1$), Cu ($3d^{10}$, $4s^1$), Ag ($4d^{10}$, $5s^1$), and Au ($5d^{10}$, $6s^1$). The total energy convergence criterion was set to be lower than e−6 eV, while the cutoff energy of 450 eV was used for the plane waves. The forces were minimized until 0.01 eV/Å, with a Gaussian smearing of 0.01 eV applied to enhance convergence and avoid fractional occupations at the Fermi level. Brillouin-zone integration’s for the gas-phase clusters were performed only at the $\Gamma$ point. In contrast, for the clusters on graphene, a **k**-mesh of $4\times 4\times 1$ was employed for the $6\times 6\times 1$ supercell.

**Figure 8:**
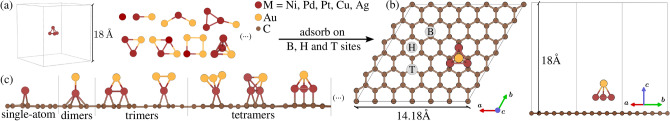
(**a**) Representation of the gas-phase cluster alloys within a cubic simulation box of size 18 Å. Gold (Au) atoms are shown in yellow, and transition metals (M = Ni, Pd, Pt, Cu, and Ag) are represented by red atoms. (**b**) Top view of the graphene supercell ($6\times 6\times 1$), highlighting the hollow (H), top (T), and bridge (B) adsorption sites. (**c**) Side view of single atoms and dimer, trimer, and tetramer cluster alloys adsorbed on the graphene surface.

To mitigate periodic replica interactions along the *a*, *b*, and *c* directions, we employed a cubic box with a size of 18 Å for the gas-phase clusters, as depicted in Fig. 8a. For the clusters supported on graphene, a $6\times 6\times 1$ supercell was used, with dimensions of 14.81Å in the *ab* plane (resulting in a system with 72 C atoms) and 18 Å of vacuum in the *c* direction, as shown in Fig. 8b. This setup is enough to avoid lateral and vertical spurious interactions between the cluster and its replicas. Since the cluster adsorption may involve interactions with two or even three atoms, we define the number of metal atoms bonded to Gr as the number of contact points or fold, e.g., tetramers can adsorb through 1-, 2-, or 3-fold coordination, as illustrated in Fig. 8c. Due to the several possible morphologies for the cluster alloys in both the gas phase and adsorbed states, many configurations were systematically generated to map the putative global minimum configurations (pGMC) in each scenario. All configurations in the gas phase, based on those employed in the adsorption process, are depicted in Figure S1 and S2 of our Supporting Information.

### Stability and structural properties

Upon obtaining the gas-phase and adsorbed pGMC for the cluster alloys, we evaluated the substrate support stability by calculating the adsorption energy ($E_{\text {ads}}$):

1 $$\begin{aligned} E_{\text {ads}}= E^{\text {full}} - E_{\text {cluster}}^\text {vac} - E_{\text {Gr}}^\text {pris}, \end{aligned}$$

where $E^\text {full}$, $E_{\text {cluster}}^{\text{vac}}$, and $E_{\text {Gr}}^{\text{pris}}$ are the energies for the entire system with the cluster alloys adsorbed, in vacuum, and for pristine graphene, respectively. Thus, negative (positive) values indicate the susceptibility to adsorption (or lack thereof) for the $\text{M}_{(\textit{n}-\textit{x})}\text{Au}_{\textit{x}}$ /Gr system. Consequently, the magnitude of the interaction energy correlates with more negative $E_{\text {ads}}$ values.

To evaluate the thermodynamic stability of the cluster alloys, i.e., their formation energies, we employed the concept of excess energy ($E_{\text {exc}}$), which indicates the favorability of mixing in both the gas phase and adsorbed situations. This approach is based on adopting the pure clusters as references, namely $\text {Au}_{x}$ and $\text {M}_{n}$. Negative $E_{\text {exc}}$ values indicate favorable mixing, while positive values suggest a segregation behavior (indicating that the mixture is not thermodynamically stable). The $E_{\text {exc}}$ is calculated as follows:

2 $$\begin{aligned} E_{\text {exc}} = E_{{\text{M}_{(\textit{n}-\textit{x})}\text{Au}_{\textit{x}} }} - \frac{(n-x)}{n}E_\text {M} - \frac{x}{n}E_{\text {Au}}, \end{aligned}$$

where $E_{\text {MAu}}$, $E_\text {M}$, and $E_{\text {Au}}$ are the energies of the binary clusters, pure M, and pure Au clusters, respectively. For the adsorbed systems, it is important to note that different adsorption configurations, i.e., varying quantities of connection points for $M_{(\textit{n}-\textit{x})}Au_{\textit{x}}$ /Gr concerning M/Gr and Au/Gr, can affect the relative stability between the binary systems and their pure cluster references. Therefore, to minimize the impact of $E_{\text {ads}}$ on $E_{\text {exc}}$, we considered similar anchoring configurations for the M/Gr and Au/Gr references.

### Electronic properties and catalytic performance

To gain a deeper understanding of the electronic properties of the systems and to elucidate the observed trends in $E_{\text {ads}}$ and $E_{\text {exc}}$, we conducted a systematic investigation of the mechanisms of ionic–covalent interactions between metal-metal atoms within the cluster and metal-C interactions between the cluster and Gr. Charge transfer and ionic character were analyzed using partial charge population via the Density Derived Electrostatic and Chemical (DDEC6)^63^ methodology. Covalent interactions were investigated through Crystal Orbital Hamilton Population (COHP)^65^ analysis, with its integration (ICOHP) indicating bond strength.

An intuitive metric for evaluating the catalytic capacity of transition metals is the *d*-band center model^84^ ($\varepsilon _{\text {d}}$). In the context of surfaces, $\varepsilon _{\text {d}}$ provides insights into scaling relations and variations in catalytic activity concerning the electronic structure of the transition-metal surface^45^. The $\varepsilon _{\text {d}}$ model is defined as the average energy ($\varepsilon$) of the occupancy density of *d*-states ($n_{d}$). The closer $\varepsilon _{\text {d}}$ is to the Fermi level ($\text {E}_\text {f}$) (high $\varepsilon _{\text {d}}$ values), the more unoccupied the *d* states of the catalyst are, enhancing the catalyst-adsorbate interaction. Nevertheless, optimal catalytic activity is reached when this interaction is of intermediate strength.

According to Sabatier’s principle, robust metal-adsorbate interactions can poison the catalyst due to the difficulty of molecular desorption, while weak interactions lead to low activity. In this context, we will employ $\varepsilon _{\text {d}}$ parameters to evaluate catalytic activity in terms of the electronic structure of transition-metal clusters, correlating them with the Sabatier’s principle. Moreover, it is well-established that gold nanostructures exhibit high catalytic activity. Therefore, the catalytic evaluation of binary clusters can be determined by comparing their $\varepsilon _{\text {d}}$ values with those of pure Au clusters. The closer the two $\varepsilon _{\text {d}}$ values, the greater the catalytic similarity.

**Figure 9:**
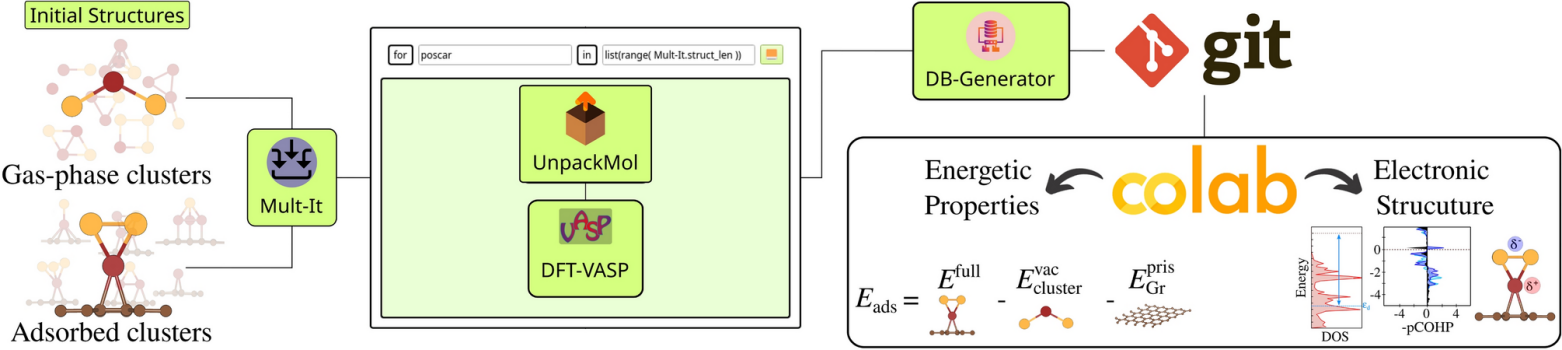
The SimStack workflow framework is used to manage the simulation protocol for calculating the energetic properties and electronic structure of unary and alloy clusters in both gas-phase and adsorbed conditions. The workflow involves several components, each serving a specific function in the process: Mult-It: manages and organizes data lists, UnpackMol: prepares configuration files for DFT calculations, DFT-VASP: carries out Density Functional Theory calculations, DB-Generator: compiles the results into a yml file. Additionally, the workflow pushes the yml file to a GitHub repository to link the generated data with a Colab notebook, where the results of the simulations are visualized.

### Scientific workflow

We generated our raw data using a set of Workflow Active Nodes (WaNos) set within the SimStack framework^55,85^, as shown in Fig. 9. We created the initial structures based on design principles (see Table S9 for the number of constructed and converged isomers), and the resulting data is available in our GitHub repository, which is generated by the integration of five distinct WaNos. Additionally, we used a Colab notebook to extract and share all post-processing data. The automation procedures aim to achieve the most stable morphologies, their corresponding energetic properties, and electronic structures.

The Mult-It WaNo generates lists and manages data within the workflow. This tool allows for creating lists containing floating-point numbers and integers and reading file name lists. In our case, it managed all POSCARs files from a given .tar archives. The processed data is then passed to the UnpackMol WaNo within the ForEach loop control. The UnpackMol WaNo decompresses configuration files and prepares them for subsequent processing within the DFT-VASP WaNo. The UnpackMol handled and prepared multiple POSCAR configurations used as input to DFT-VASP. The integration is critical in streamlining the data preparation process for density functional theory (DFT) calculations. The DFT-VASP WaNo is developed to facilitate the submission, monitoring, and retrieval of Density Functional Theory (DFT) calculations data using the Vienna *Ab initio* Simulation Package (VASP)^79,80^. This WaNo is used to simplify the optimization of such calculations, making it accessible to researchers regardless of their familiarity with the complex functionalities of VASP. The DB-Generator WaNo is used to generate files in yml format that comprehensively detail the total energies obtained from DFT-VASP calculations and key other parameters extracted from the OUTCAR files, which at the same time tracks the calculation records of a specific POSCAR configuration. These files were essential for the subsequent stages of our workflow, as they were automatically pushed to a GitHub repository and imported into a Colab notebook, which functions as the dataset for evaluating their stability and catalytical properties.

The primary goal of this workflow was to capture a complex protocol’s components and automate its execution. This workflow guaranteed scalability, reproducibility, transferability, and flexibility of the presented results by adhering to the FAIR (Findable, Accessible, Interoperable, and Reusable) and TRUE (Transparent, Reproducible, Usable by Others, and Extensible) data principles^55,85^, enhancing the reliability and accessibility of our findings. All results presented are based on the raw data generated using all four WaNos. Further technical details about the workflow, Colab notebook, and data are shared on our GitHub repository.

## Supplementary Information

Supplementary Information.

## Data Availability

The authors confirm that the data supporting this study’s findings are available in the article and the materials and under request. The Workflow is also openly public in the Au-alloy-supported-on-graphene repository at https://github.com/KIT-Workflows/Au-alloy-supported-on-graphene.
