# Supplementary material for: Workflow-driven catalytic modulation from single-atom catalysts to Au–alloy clusters on graphene
Source: Sci Rep. 2025 Jan 14;15:1939. doi: 10.1038/s41598-025-85891-6 (PMC11733030; doi:10.1038/s41598-025-85891-6)
Supplement: Supplementary file 1 — Supplementary Information. [file 41598_2025_85891_MOESM1_ESM.pdf]

Workflow-Driven Catalytic Modulation from Single-Atom Catalysts to Au–Alloy Clusters on Graphene

Gabriel Reynald Da Silva[1], João Paulo Cerqueira Felix[2], Celso R. C. Rêgo[5,*], Alexandre C. Dias[4], Carlos Maciel de O. Bastos[4], Maurício J. Piotrowski[3], and Diego Guedes-Sobrinho[1]

[1]Federal University of Paraná, Department of Chemistry, Curitiba, 81531-980, Brazil
[2]Institute of Physics "Armando Dias Tavares", Rio de Janeiro, 20550-900, Brazil
[3]Federal University of Pelotas, Department of Physics, Pelotas, 96010-900, Brazil
[4]University of Brasília, Institute of Physics and International Center of Physics, Brasília, 70919-970, Brazil
[5]Karlsruhe Institute of Technology, Institute of Nanotechnology Hermann-von-Helmholtz-Platz, Karlsruhe, 76021, Germany
[*]celso.rego@kit.edu

1 Previously studies comparison

Several of the systems investigated in this work, particularly the monometallic ones, have been previously studied. To verify the reliability of our methodology, we evaluted the binding energy (E_b) for clusters in the gas phase under vacuum conditions and the adsorption energy (E_ads) for clusters adsorbed on graphene, allowing direct comparison with both theoretical and experimental data reported in the literature, as detailed in Tables S1–S5. The parameters were calculated using the following expressions:

$$E_\mathrm{b} = E_\mathrm{cluster} - nE_\mathrm{atom}, \quad (1)$$

where E_cluster, E_atom, and n denote the energy of the cluster, the energy of an isolated atom, and the number of atoms in the cluster, respectively; and

$$E_\mathrm{ads} = E^\mathrm{full} - E^\mathrm{vac}_\mathrm{cluster} - E^\mathrm{pris}_\mathrm{Gr}, \quad (2)$$

where E^full, $E^\mathrm{vac}_\mathrm{cluster}$, and $E^\mathrm{pris}_\mathrm{Gr}$ correspond to the energies of the complete system with the cluster adsorbed, the cluster in vacuum, and pristine graphene, respectively.

	E_b for clusters on vac. (eV/n)		
	This work	References	Experimental
Ni_2	−1.47	−1.46[1], −1.41[2]	−1.60[3]
Ni_3	−1.81	−1.85[1], −1.81[2]	
Ni_4	−2.11	−2.18[1], −2.14[2]	
Pd_2	−0.67	−0.68[1], −0.66[2]	−0.66[4]
Pd_3	−1.30	−1.26[1], −1.28[2]	
Pd_4	−1.71	−1.73[1], −1.69[2]	
Pt_2	-1.90	−1.88[1], −1.90[2]	−1.86[5]
Pt_3	−2.52	−2.45[1], −2.46[2]	
Pt_4	−2.83	−2.76[1], −2.76[2]	
Cu_2	−1.14	−1.14[1], −1.12[6]	−1.11[7]
Cu_3	−1.24	−1.24[1], −1.26[6]	
Cu_4	−1.60	−1.62[1], −1.63[6]	
Ag_2	−0.89	−0.89[1]	−0.89[7]
Ag_3	−0.89	−0.89[1]	
Ag_4	−1.18	−1.19[1]	
Au_2	−1.18	−1.16[1]	−1.16[8]
Au_3	−1.25	−1.23[1]	
Au_4	−1.57	−1.57[1]	

Table S1 – Bond energy (E_b) calculated for the most stable structures of unary Ni_n, Pd_n, Pt_n, Cu_n, Ag_n, and Au_n (with $n = 2-4$) and compared to previously theoretical and experimental works.

	Site	E_{ads} (eV)	References	Deviation(%)
Au_{1}/Gr	T	0.38	0.42[1], ~0.40[9]	9.52[1], ~5.00[9]
Ag_{1}/Gr	T	0.23	0.28[1],~0.25[9]	17.86[1], ~8.00[9]
Cu_{1}/Gr	T	0.44	0.58[10], 0.55[1], ~0.50[9]	20.00[1], 12.00[9]
Ni_{1}/Gr	H	1.57	1.81[2], 1.60[1], ~1.60[9]	13.26, 1.88[1,9]
Pd_{1}/Gr	B	1.31	1.34[2], 1.39[1], ~1.40[9]	2.24[2], 5.75[1], 6.43[9]
Pt_{1}/Gr	B	1.93	1.88[2], 1.97[11], 1.92[1], ~1.90[9]	2.66[2], 2.03[11], 0.52[1], ~1.58[9]

Table S2 – The most stable single-atom configurations adsorbed on graphene, indicating their preferred adsorption sites—top (T), bridge (B), or hollow (H)—along with the corresponding adsorption energy (E_{ads}). The deviations of these values from previously reported results are also provided for comparison.

	Site	E_{ads} (eV)	References	Deviation (%)
$Au_{2/}Gr$	T	0.81	0.82[1]	1.22
$Ag_{2/}Gr$	T	0.37	0.43[1]	13.95
$Cu_{2/}Gr$	B	0.82	0.88[1]	6.82
$Ni_{2/}Gr$	H	1.57	1.66[2]	5.42
$Pd_{2/}Gr$	BB	1.86	1.80[2]	3.33
$Pt_{2/}Gr$	B	1.53	1.42[2]	7.75

Table S3 – The most stable unary dimers configurations adsorbed on graphene, indicating their preferred adsorption sites—top (T), bridge (B), or hollow (H)—along with the corresponding adsorption energy (E_{ads}). The deviations of these values from previously reported results are also provided for comparison.

	Site	E_{ads} (eV)	References	Deviation (%)
$Au_{3/}Gr$	–	0.94	1.06[1]	11.30
$Ag_{3/}Gr$	T	0.77	0.99[1]	22.22
$Cu_{3/}Gr$	BB	1.34	1.49[1]	10.10
$Ni_{3/}Gr$	BB	2.02	2.19[1]	7.76
$Pd_{3/}Gr$	BBB	1.43	1.67[1]	14.37
$Pt_{3/}Gr$	BB	2.12	2.13[1]	0.47

Table S4 – The most stable unary trimers configurations adsorbed on graphene, indicating their preferred adsorption sites—top (T), bridge (B), or hollow (H)—along with the corresponding adsorption energy (E_{ads}). The deviations of these values from previously reported results are also provided for comparison.

	Site	E_{ads} (eV)	References	Deviation (%)
$Au_{4/}Gr$	T	1.20	1.22[1]	1.63
$Ag_{4/}Gr$	T	0.73	0.85[1]	14.12
$Cu_{4/}Gr$	B	1.05	1.15[1]	8.69
$Ni_{4/}Gr$	BBB	2.47	2.58[1]	4.26
$Pd_{4/}Gr$	BB	1.46	1.66[1]	12.05
$Pt_{4/}Gr$	BB	2.26	2.32[1]	2.59

Table S5 – The most stable unary tetramers configurations adsorbed on graphene, indicating their preferred adsorption sites—top (T), bridge (B), or hollow (H)—along with the corresponding adsorption energy (E_{ads}). The deviations of these values from previously reported results are also provided for comparison.

2 Adsorption energy

The superior adsorption of alloys on graphene is consistently observed in comparison to unary cluster isomers with similar adsorption configuration morphology. One observes that positioning Au at exposed sites significantly increases E_{ads}, as demonstrated in Table S6–S8.

	E_{ads} (eV)				
	Ni	Pd	Pt	Cu	Ag
M_1Au_1	-1.65	-1.09	-1.75	-1.13	-0.59
M_2	-1.16	-0.93	-1.32	-0.82	-0.37

Table S6 – Adsorption energy of adsorbed M_1Au_1 dimers alloys pGMC compared to M_2 unary cluster isomer with similar morphology.

	E_{ads} (eV)				
	Ni	Pd	Pt	Cu	Ag
M_2Au_1	-2.70	-1.73	-2.38	-1.36	-0.71
M_3	-2.01	-1.31	-2.12	-1.34	-0.77
M_1Au_2	-1.54	-1.26	-1.86	-1.32	-0.80
M_3	-1.46	-1.02	-1.81	-1.24	-0.77

Table S7 – Adsorption energy of adsorbed $M_{(3\text{-}x)}Au_x$ trimers alloys pGMC compared to M_3 unary cluster isomer with similar morphology.

	E_{ads} (eV)				
	Ni	Pd	Pt	Cu	Ag
M_3Au_1	-2.48	-1.85	-2.16	-1.09	-0.72
M_4	-2.47	-1.33	-2.12	-1.05	-0.68
M_2Au_2	-1.54	-1.67	-2.16	-1.14	-0.76
M_4	-1.26	-1.46	-2.12	-1.05	-0.68
M_1Au_3	-1.86	-1.37	-1.87	-1.29	-0.92
M_4	-0.85	-0.44	-1.76	-1.05	-0.73

Table S8 – Adsorption energy of adsorbed $M_{(4\text{-}x)}Au_x$ tetramers alloys pGMC compared to M_4 unary cluster isomer with similar morphology.

3 Relative energy for all converged isomers

Given the possibility of numerous isomers, a significant challenge is mapping the potential energy surface to find the lowest energy structures (global minima) and their associated meta–stable states (local minima). As the lowest energy structures may vary depending on the theoretical level or methods used, the ground states found are termed candidates for the putative global minima configuration (pGMC). The relative energy ($E_{\rm rel}$) is a way to analyze the relative stability of possible isomers compared to the ground state. It is calculated by:

$$E_{\rm rel} = E_{\rm isomer} - E_{\rm pGMC},$$

where $E_{\rm pGMC}$ is the energy of the found ground state and $E_{\rm isomer}$ is the energy of another converged isomer, considered a meta–stable state. $E_{\rm rel}$ represents the energy difference between the global minimum and a local minimum on the potential energy surface. It is important to note that $E_{\rm rel}$ is not the energy required for the transition from one configuration to another, as such a process requires overcoming an energy barrier that has not been calculated here. Moreover, this mapping is related to the probability and distribution of forming a particular isomer. Figures S1 and S2 illustrate the contribution of this work to the discussed problem.

This work employed design principles methodology for the initial construction of isomers, considering all possible geometries and using symmetry operations to discard redundant isomers. Table S9 shows the number of constructed and converged isomers. It is important to note that some initial configurations are unstable and converge to local or global minima, thus being disregarded.

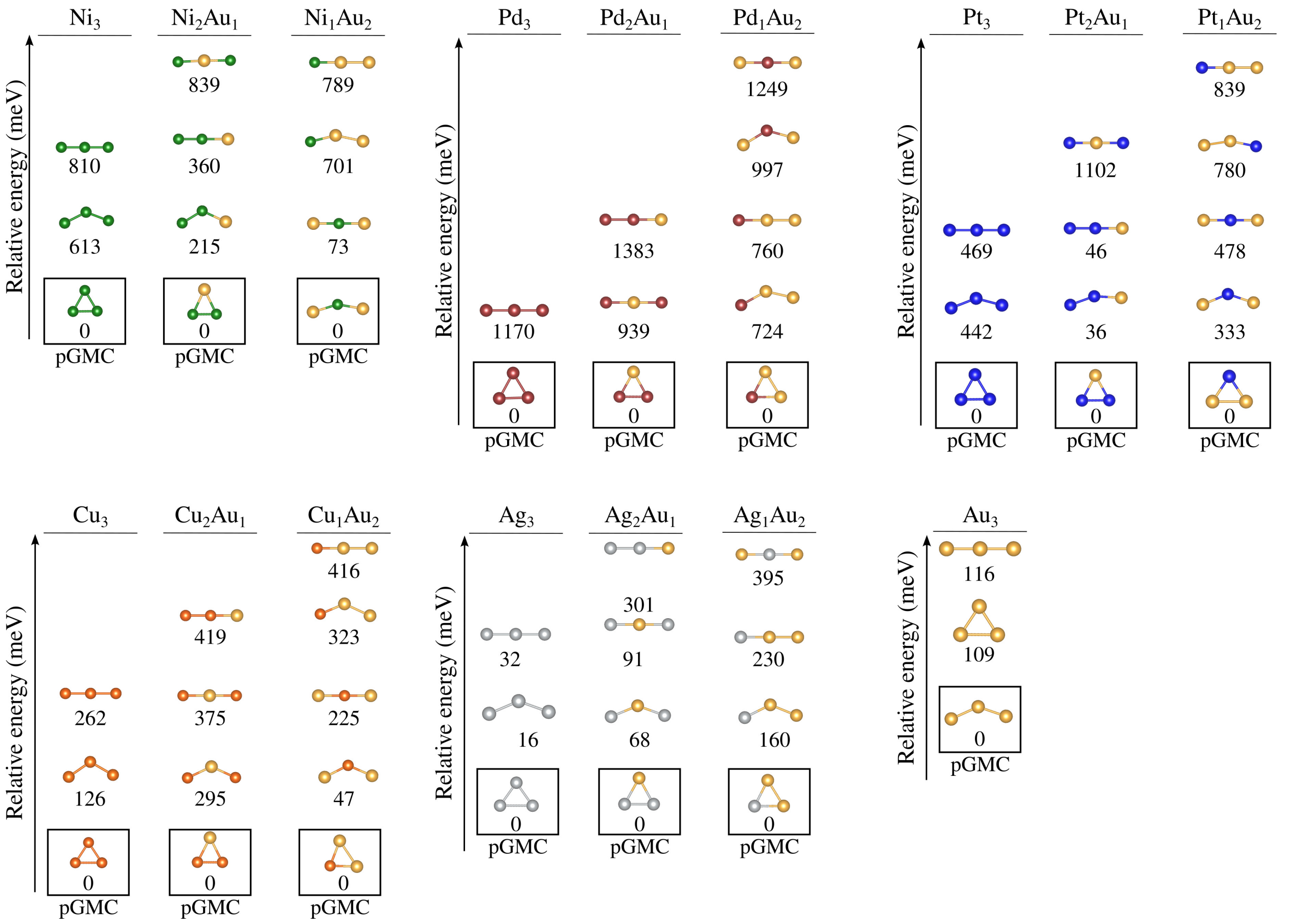

Figure S1 – The relative energy ($E_{\rm rel}$) among converged isomers of unary and alloys trimers (n = 3) with respect to the ground state (pGMC), expressed in meV**.**

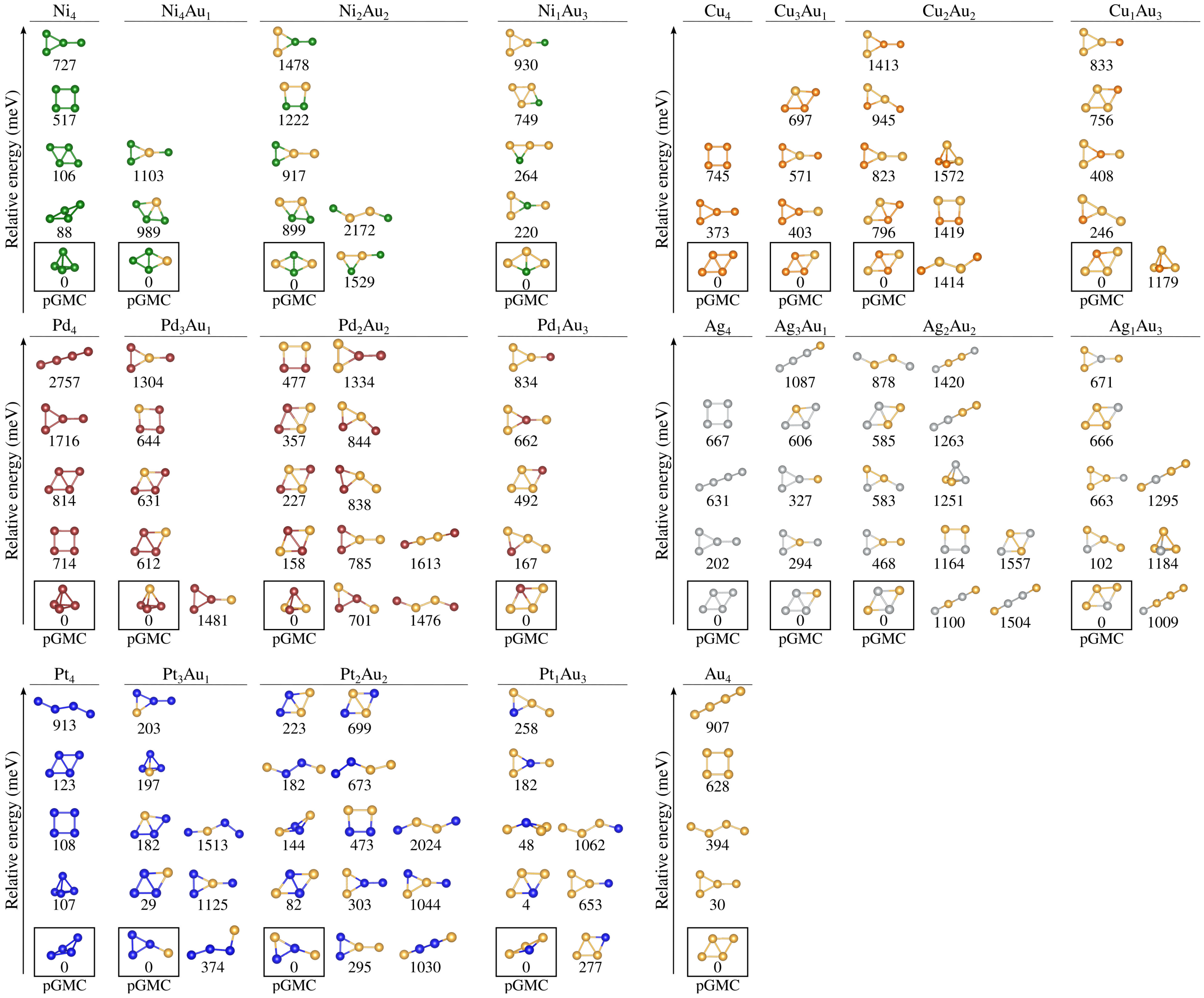

Figure S2 – The relative energy (E_{rel}) among converged isomers of unary and alloys tetramers ($n = 4$) with respect to the ground state (pGMC), expressed in meV**.**

		Constructed isomers	Converged isomers
vac.	trimers ($n = 3$)	68	59
	tetramers ($n = 4$)	236	136

Table S9 – The number of constructed isomers and the metastable states converged for unary and alloys clusters in gas–phase on vacuum.

4 Structural parameters

To analyze the structural characteristics, some parameters that are widely employed in theoretical studies of clusters and NC's were adopted, particularly the effective coordination number (ECN), the average bond length (d_{av}), and the radius of gyration (R_{g}). The ECN is an approach to analyze distances and coordination numbers applied to the solid state, which deviates from classical inorganic analyses, that accounting for degrees of disorder and local distortions.[12,13] For this, the distance between atoms i and j is given as d_{ij} and the average distance for each atom i as d^i_{av}, where

$$d^i_{av} = \frac{\sum_j d_{ij} exp\left[1 - \left(\frac{d_{ij}}{d^i_{av}}\right)^6\right]}{\sum_j exp\left[1 - \left(\frac{d_{ij}}{d^i_{av}}\right)^6\right]}, \tag{3}$$

in which a self-consistent calculation iterate $d^i_{av(initial)}$ and $d^i_{av(final)}$ until $|d^i_{av(final)} - d^i_{av(initial)}| < 10^{-4}$. Once d^i_{av} is obtained, the effective coordination number of each atom i (ECN_i) can be calculated by

$$ECN_i = \sum_j exp\left[1 - \left(\frac{d_{ij}}{d^i_{av}}\right)^6\right]. \tag{4}$$

To obtain the averages of the parameters d^i_{av} and ECN_i, the following equations are applied, respectively:

$$d_{av} = \frac{1}{n}\sum_{i=1}^{n} d^i_{av} \quad \text{and} \quad ECN = \frac{1}{n}\sum_{i=1}^{n} ECN_i, \tag{5}$$

where n is the number of atoms that compose the cluster. On the other hand, R_{g} is physically defined as the radial distance between the moment of inertia and the center of mass, and is mathematically described as the root mean square of the distances r of each atom i from the center of mass of the system.

$$R_g = \sqrt{\frac{1}{n}\sum_{i=1}^{n} r_i^2}, \tag{6}$$

in which n is the number of atoms in the cluster.

In order to characterize the structural properties of the adsorbed systems, d_{av}, ECN, and R_{g} were recalculated to assess surface effects on these properties. Additionally, the distance between the cluster and graphene (d_{L}) was evaluated. All these results, as a function of the composition x of the $M_{(n\text{-}x)}Au_x$ clusters, are presented in Figure S3.

As illustrated in Figure S3(a), the bond distances evaluated for unary and binary clusters indicate that, in the adsorbed state, there is an increase in the d_{av} as a general behavior. This increase is particularly notable in the case of the Pd_2/Gr and Pd_3/Gr (around 0.10 Å), which is due to the fact that their pGMCs have more contact points with Gr (2 and 3, respectively) compared to other pure clusters of the same atomicity. This demonstrates that the M−M bond length is considerably affected when both M atoms are bonded to Gr. For alloys, it is observed that for compositions with few Au atoms, adsorption has a smaller effect on the increase of d_{av}. However, for higher values of x, this effect is more pronounced. This behavior can be attributed to the fact that, despite the increase in M−M bond lengths upon adsorption, all M−Au bonds become shorter, compensating for both and minimizing the impact on d_{av}. However, the Au−Au bond length also increases upon adsorption, which results in a lack of compensation at higher x compositions, thereby exerting a more pronounced impact on d_{av}.

The variation of ECN for adsorbed clusters, depicted in Figure S3(b), is attributed to the morphological change observed when comparing the cluster in the gas–phase on vacuum to adsorbed system. In general, there is an increase in coordination, as some less coordinated configurations (e.g., diamond) in gas–phase become more compact configurations (e.g., tetrahedron) when adsorbed. However, no cluster with a compact morphology in gas–phase becomes planar upon adsorption. Moreover, the morphologies of unary and binary tetramers can be identified as tetrahedral, diamond, or capped-triangular if the ECN is around 3.00, 2.50, and 2.00, respectively.

Regarding R_{g}, the adsorption of the cluster promotes a subtle increase that follows the trend observed for d_{av}, as shown in Figure S3(c). Only the Ni_1Au_2 system is an exception to this behavior, as it changes its angular configuration in the gas phase, which promotes a larger R_{g}, to a triangular one upon adsorption.

The layer distance d_{L}, shown in Figure S3(d), is largely contingent upon the site at which the M atom is inserted. Only Ni systems are capable of adsorbing in hollow (H) sites, and for those that do, the distance to Gr is approximately 1.75 Å. A

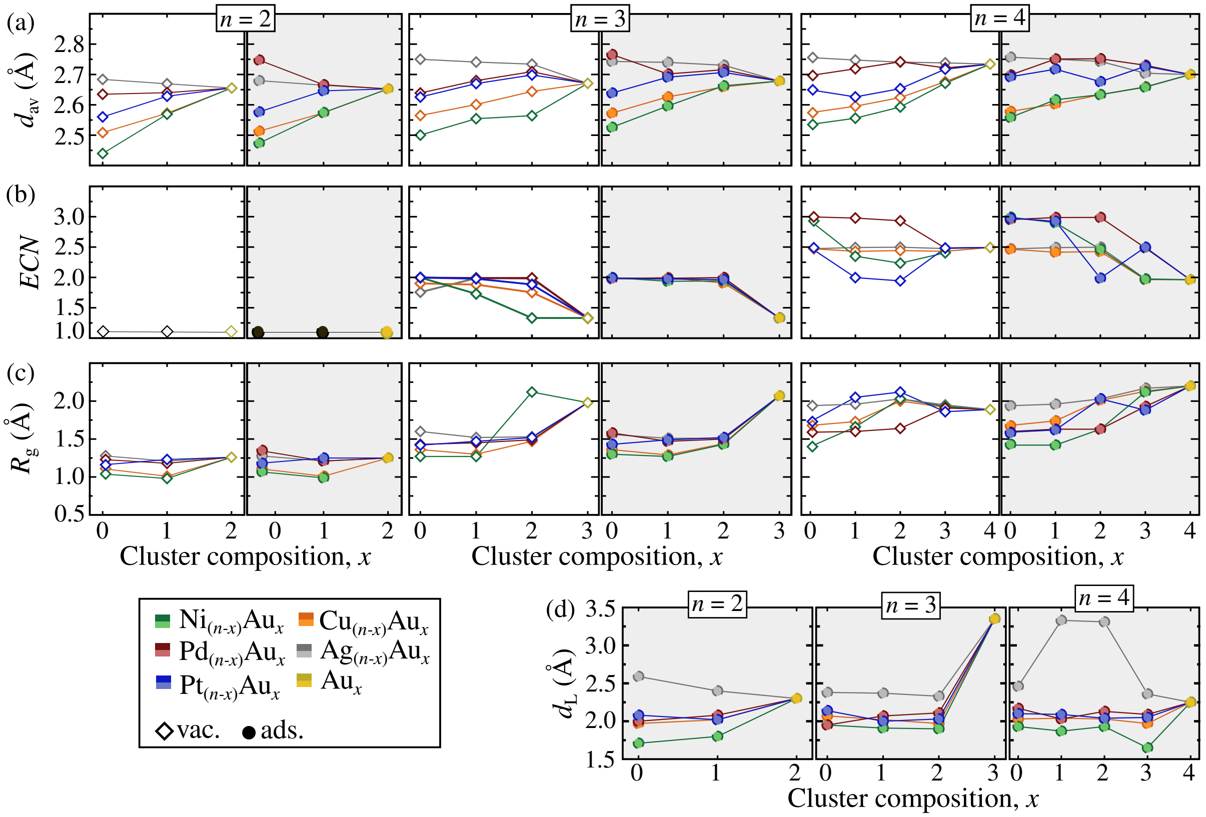

Figure S3 – (a) The average bond length (d_{av}), (b) the effective coordination number (*ECN*), (c) the radius of gyration (R_g) and (d) the distance from cluster to graphene (d_L) are shown for dimers ($n = 2$), trimers ($n = 3$) and tetramers ($n = 4$) as function of composition x. Adsorbed clusters are highlighted by a gray background.

greater variation in d_L is observed for systems that adhere to the bridge (B) site compared to those, which are related to the coordination of M atoms. If the M atoms directly bonded to C are highly coordinated with other metals in the cluster, the M−C bond length will increase slightly, which will result in the observed phenomenon. Therefore, the d_L of clusters adsorbed at B sites varies between 1.90 and 2.30 Å. For d_L above 2.50 Å, the distance remains associated with adsorption at top (T) sites. Furthermore, there is the possibility of no contact points between the cluster and Gr (0–fold configuration), as observed for Au_3, Ag_1Au_3, and Ag_2Au_2, where d_L reaches 3.50 Å. Notwithstanding the substantial disparity in d_L between adsorption at T and 0–fold sites, E_{ads} exhibits minimal variation. This also indicates that both configurations are associated with a physisorption process.

5 Charge

Aiming to evaluate the charge transfer between metal atoms and partial charges at specific sites, we calculeted the charge population through the Density Derived Electrostatic and Chemical (DDEC6) method[14] and the Bader analysis[15] for unary and alloy clusters in the gas–phase under vacuum and adsorbed on graphene, which is depicted in Figures S3–S5.

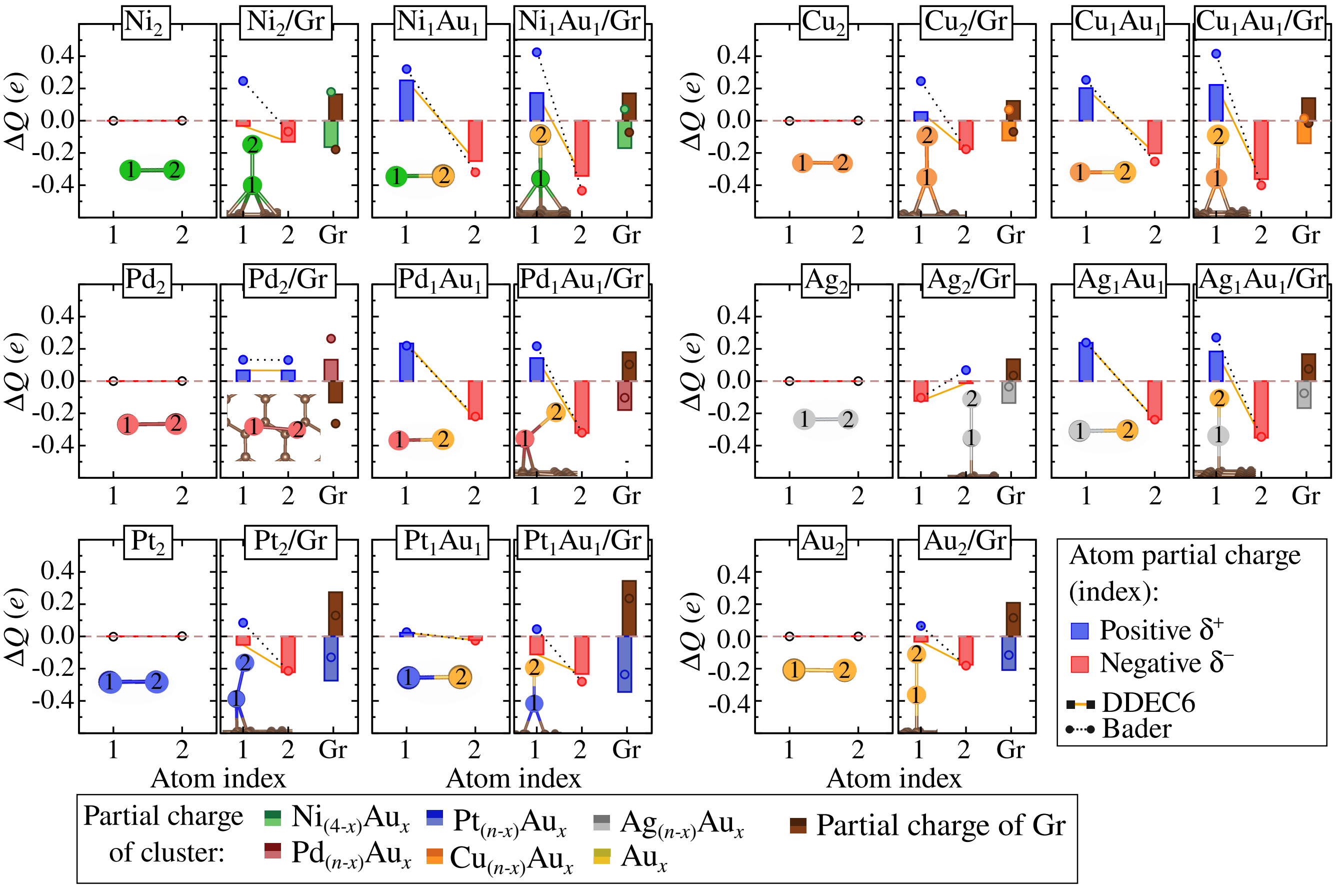

Figure S4 – Partial charge (ΔQ) for each atom of unary and alloys dimers (n = 2) in gas–phase on vacuum and adsorbed in graphene (Gr) obtained by DDEC6 and Bader methodology, with positive and negative partial charges are reddish and blueish bars, respectively. Total ΔQ of clusters is also indicate by green, dark-red, dark-blue, orange, silver, and golden bars for Ni, Pd, Pt, Cu, Ag, and Au systems, respectively. Total ΔQ of Gr is indicate by brown bars.

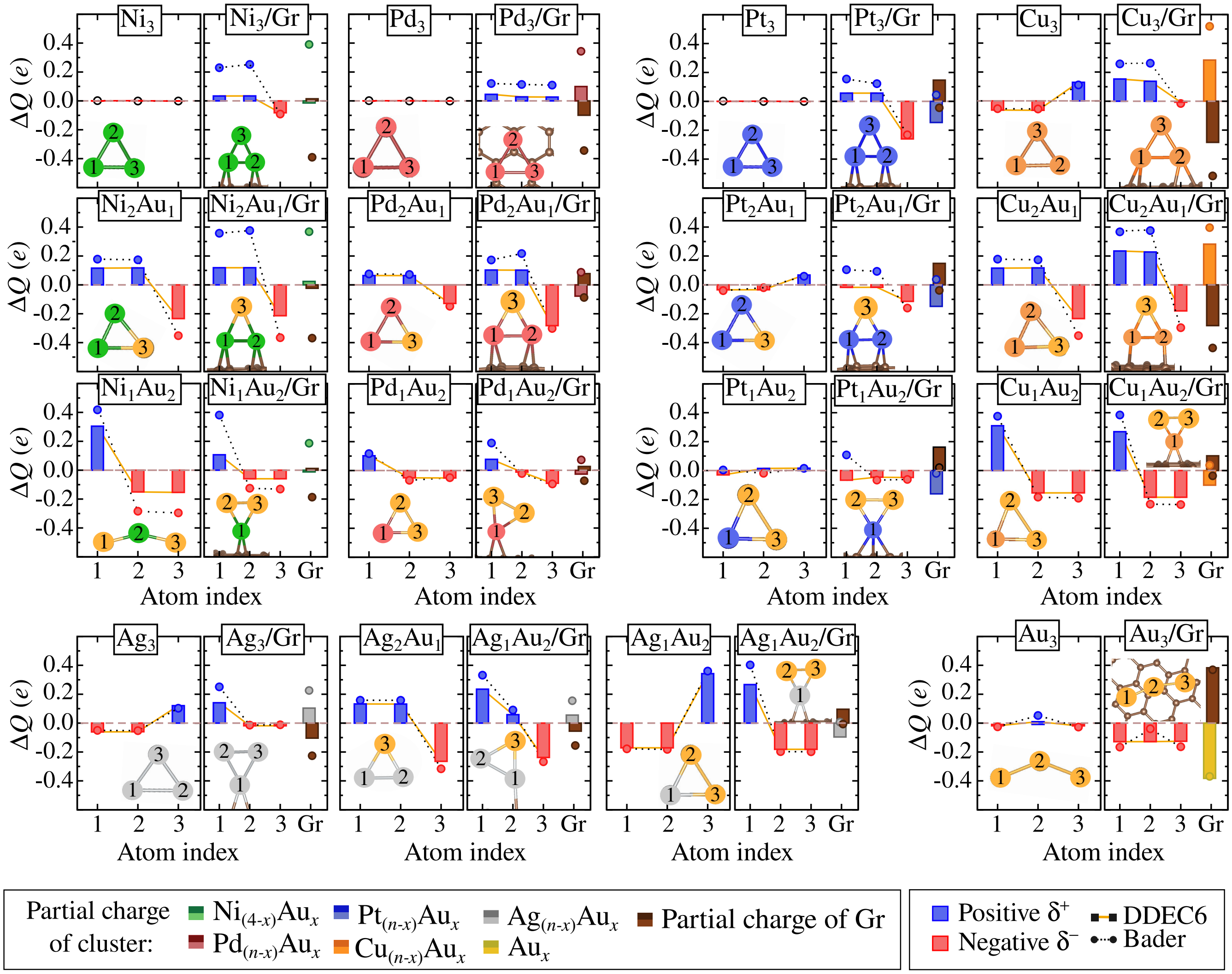

Figure S5 – Partial charge (ΔQ) for each atom indexed of unary and binary trimers (n = 3) in gas–phase on vacuum and adsorbed in graphene (Gr) obtained by DDEC6 and Bader methodology, with positive and negative partial charges are reddish and blueish. Total ΔQ of clusters is also indicate by green, dark-red, dark-blue, orange, silver, and golden bars for Ni, Pd, Pt, Cu, Ag, and Au systems, respectively. Total ΔQ of Gr is indicate by brown bars.

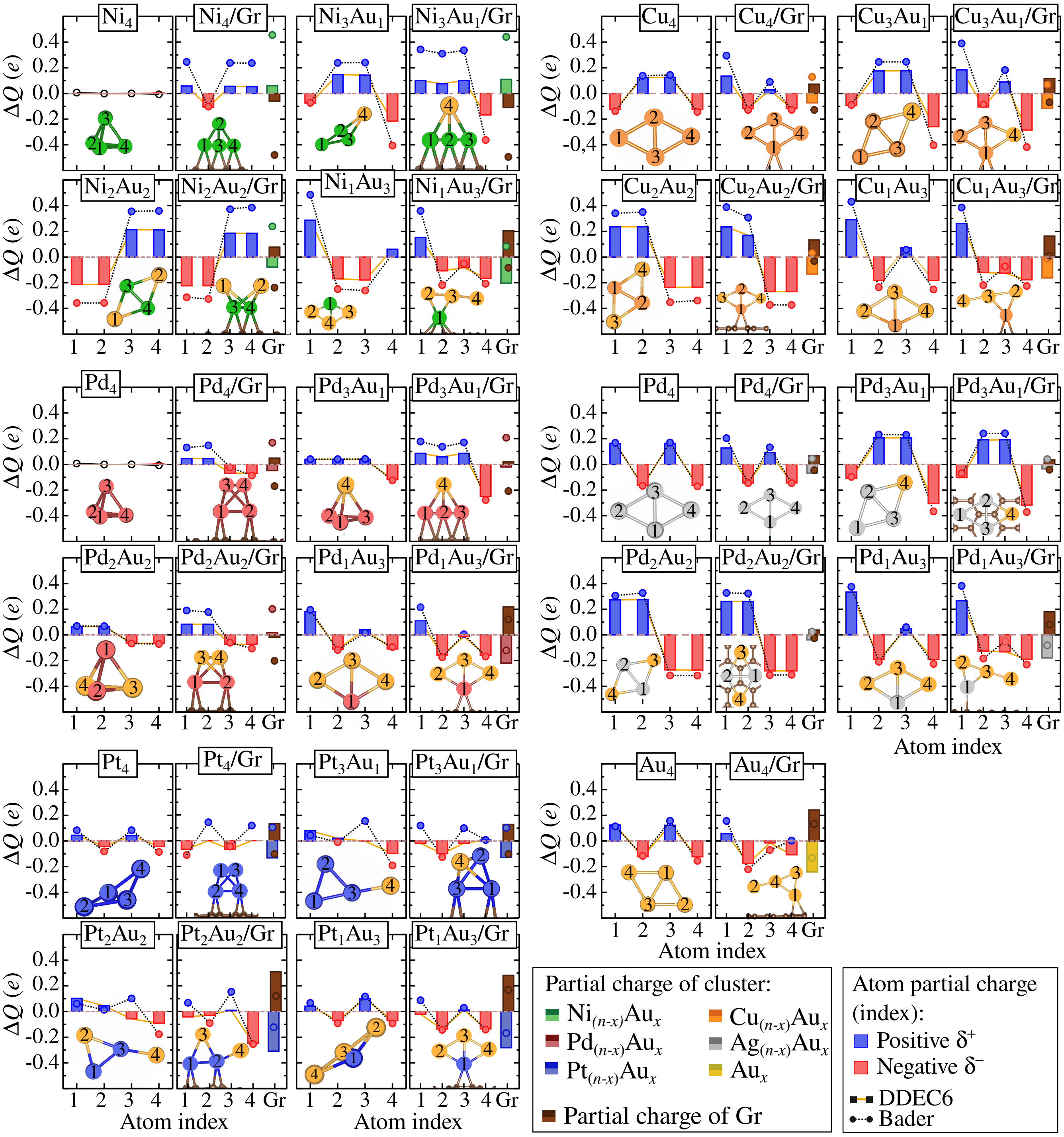

Figure S6 – Partial charge (ΔQ) for each atom indexed of unary and binary tetramers ($n = 4$) in gas–phase on vacuum and adsorbed in graphene (Gr) obtained by DDEC6 and Bader methodology, with positive and negative partial charges are reddish and blueish. Total ΔQ of clusters is also indicate by green, dark-red, dark-blue, orange, silver, and golden bars for Ni, Pd, Pt, Cu, Ag, and Au systems, respectively. Total ΔQ of Gr is indicate by brown bars.

6 COHP

To evaluate the covalent interactions between metal–metal and metal–carbon bonds, we employed the Crystal Orbital Hamilton Population (COHP) analysis[16], which provides an indication of bond strength ($|\mathcal{F}|$) through the integration of COHP (ICOHP). Figures S6–S15 present these results for unary and alloy clusters in the gas phase and adsorbed on graphene.

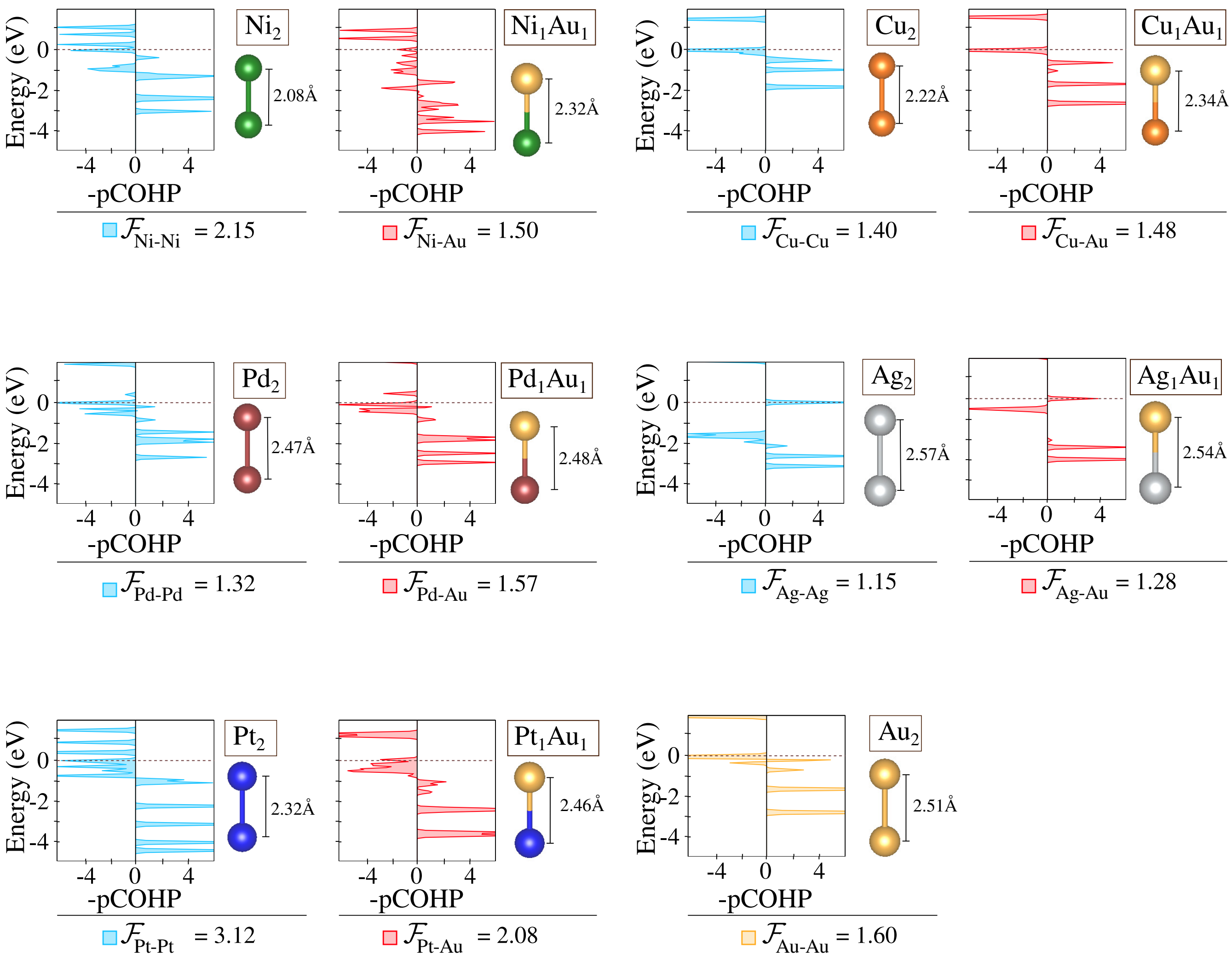

Figure S7 – Crystal Orbital Hamilton Population (COHP) analysis for unary and binary dimers (*n* = 2) clusters in the gas phase for M–M and M–Au interactions, which $|\mathcal{F}_{\text{M-Au,Au-Au,M-M}}|$ as the covalent bond strength through the integration of COHP (ICOHP) values provided in eV and normalized by equivalent bonds. The asterisk (*) indicates configurations where a given element adopts a non-similar structural arrangement.

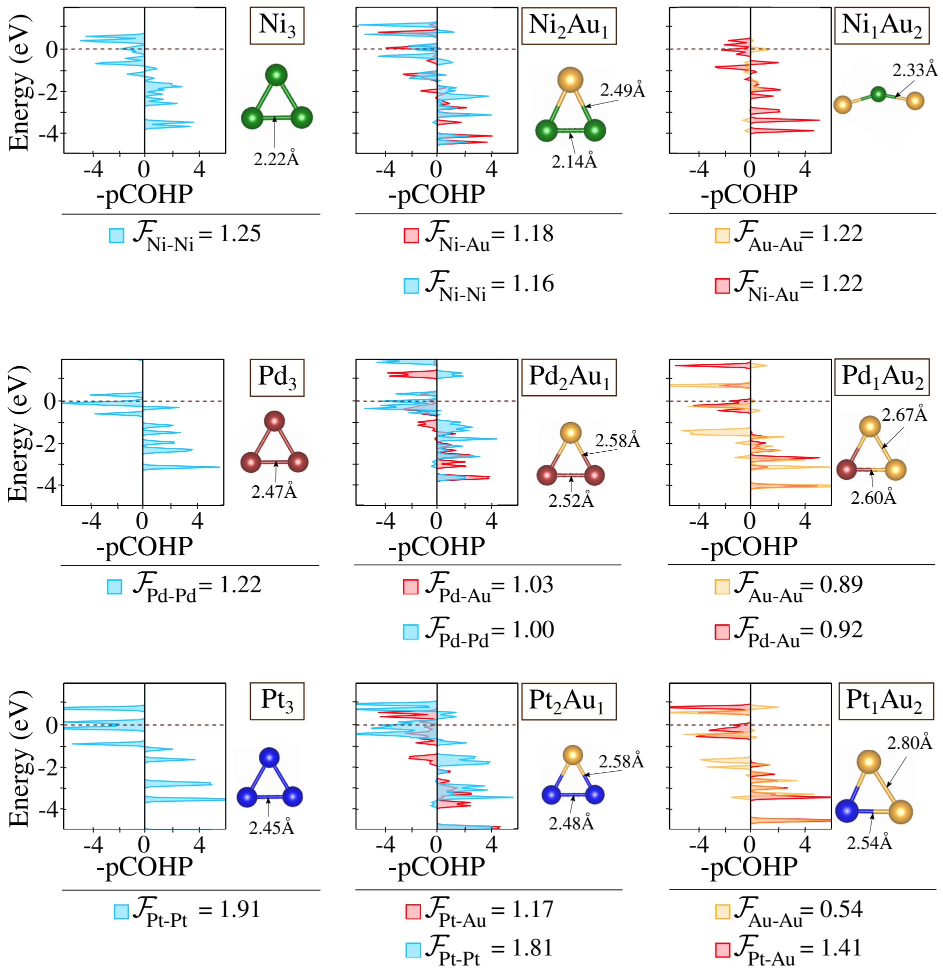

Figure S8 – Crystal Orbital Hamilton Population (COHP) analysis for unary and binary trimers (n = 3) clusters in the gas phase for M–M and M–Au interactions, which $|\mathcal{F}_{\text{M-Au,Au-Au,M-M}}|$ as the covalent bond strength through the integration of COHP (ICOHP) values provided in eV and normalized by equivalent bonds. The asterisk (*) indicates configurations where a given element adopts a non-similar structural arrangement.

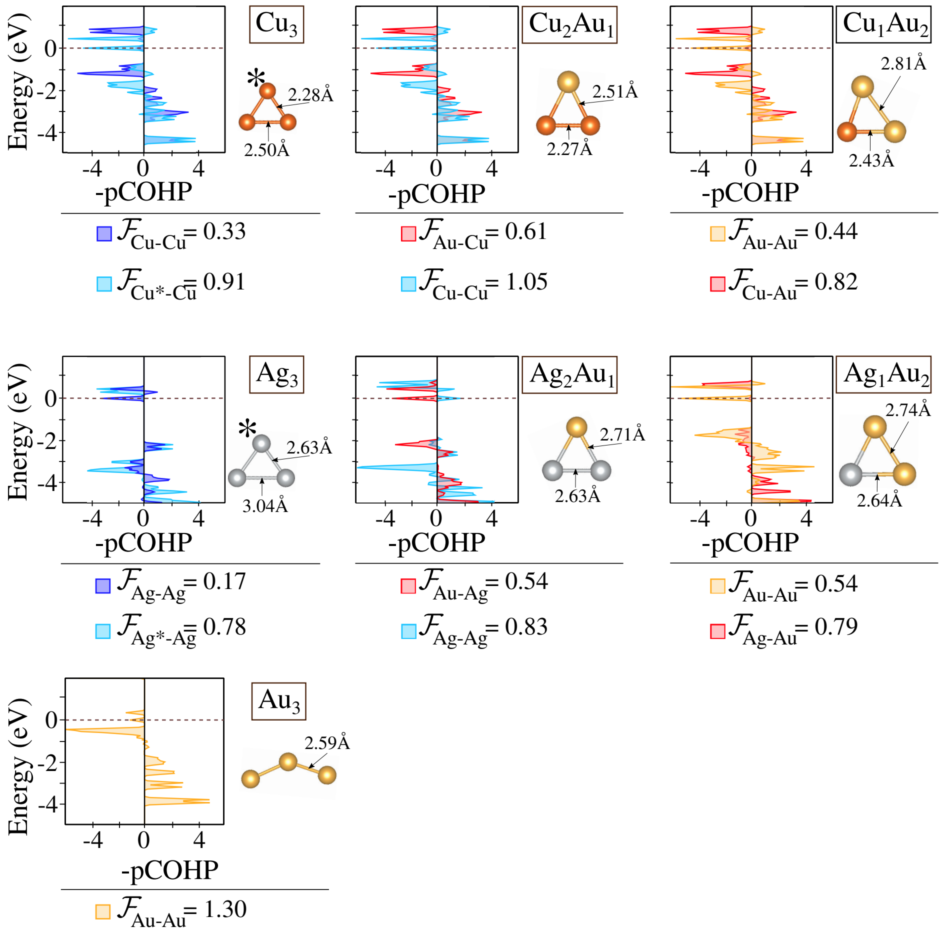

Figure S9 – Crystal Orbital Hamilton Population (COHP) analysis for unary and binary trimers (n = 3) clusters in the gas phase for M−M and M−Au interactions, which $|\mathcal{F}_{\text{M-Au,Au-Au,M-M}}|$ as the covalent bond strength through the integration of COHP (ICOHP) values provided in eV and normalized by equivalent bonds. The asterisk (*) indicates configurations where a given element adopts a non-similar structural arrangement.

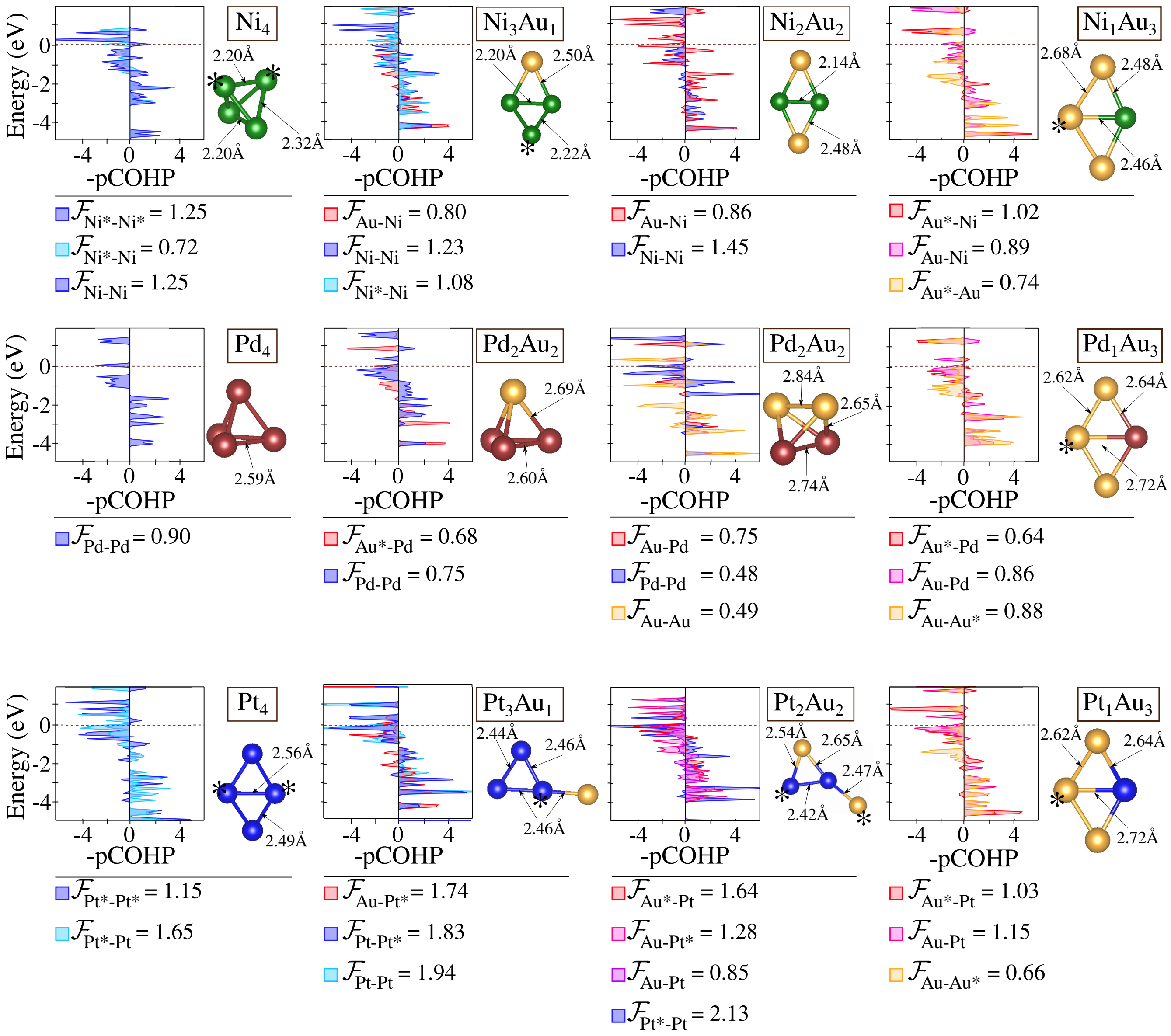

Figure S10 – Crystal Orbital Hamilton Population (COHP) analysis for unary and binary tetramers (*n* = 4) clusters in the gas phase for M−M and M−Au interactions, which $|\mathcal{F}_{M\text{-}Au,Au\text{-}Au,M\text{-}M}|$ as the covalent bond strength through the integration of COHP (ICOHP) values provided in eV and normalized by equivalent bonds. The asterisk (*) indicates configurations where a given element adopts a non-similar structural arrangement.

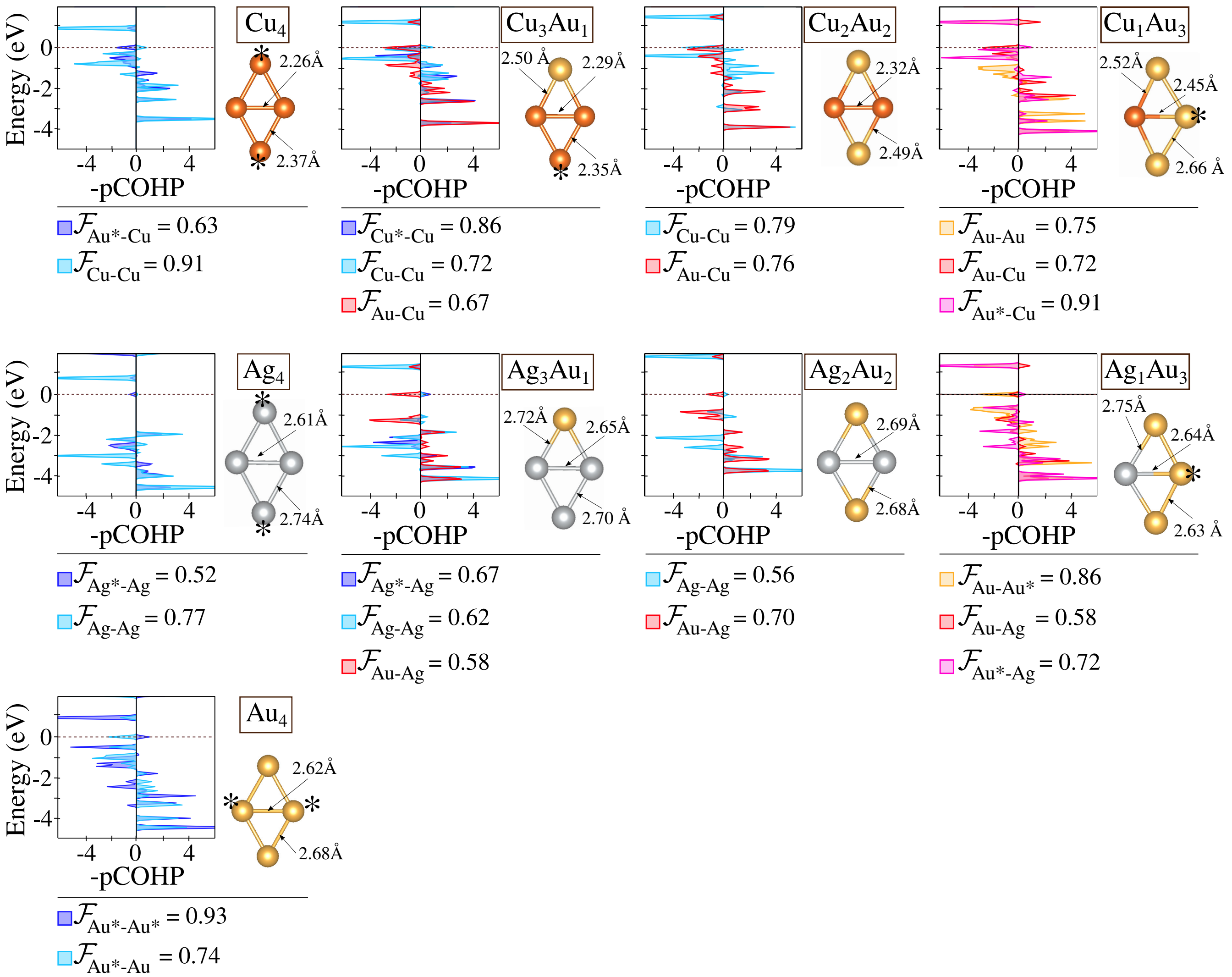

Figure S11 – Crystal Orbital Hamilton Population (COHP) analysis for unary and binary tetramers (n = 4) clusters in the gas phase for M−M and M−Au interactions, which $|\mathcal{F}_{\mathbf{M\text{-}Au,Au\text{-}Au,M\text{-}M}}|$ as the covalent bond strength through the integration of COHP (ICOHP) values provided in eV and normalized by equivalent bonds. The asterisk (*) indicates configurations where a given element adopts a non-similar structural arrangement.

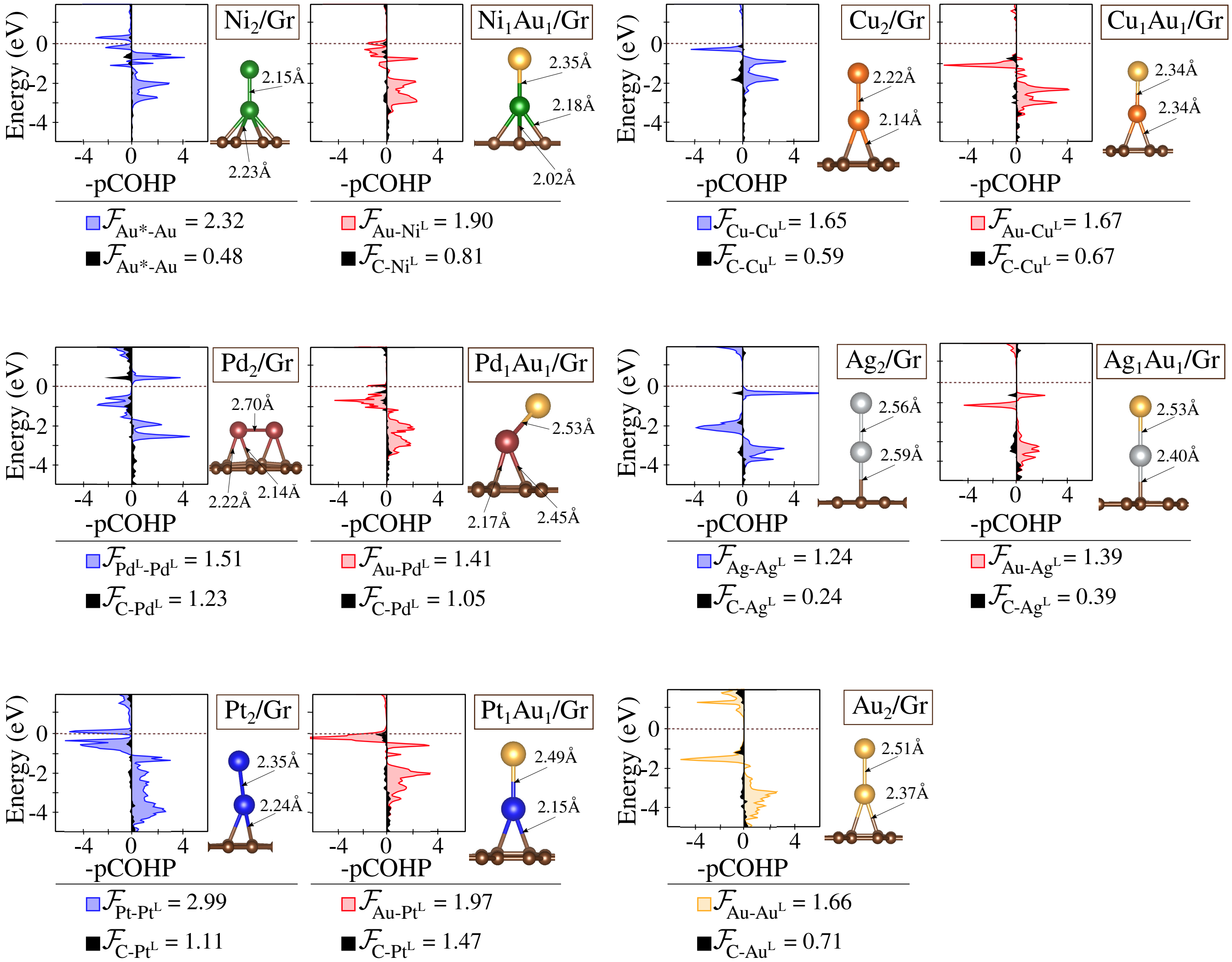

Figure S12 – Crystal Orbital Hamilton Population (COHP) analysis for unary and binary dimers (n = 2) clusters adsorbed on graphene (Gr) for M−M, M−Au, Au−Au, and C−M interactions, which $|\mathcal{F}_{M\text{-}Au,Au\text{-}Au,M\text{-}M,C\text{-}M}|$ as the covalent bond strength through the integration of COHP (ICOHP) values provided in eV and normalized by equivalent bonds. The ^{L} notation denotes the metal atoms bonded to Gr, while the asterisk (*) indicates configurations where a given element adopts a non-similar structural arrangement.

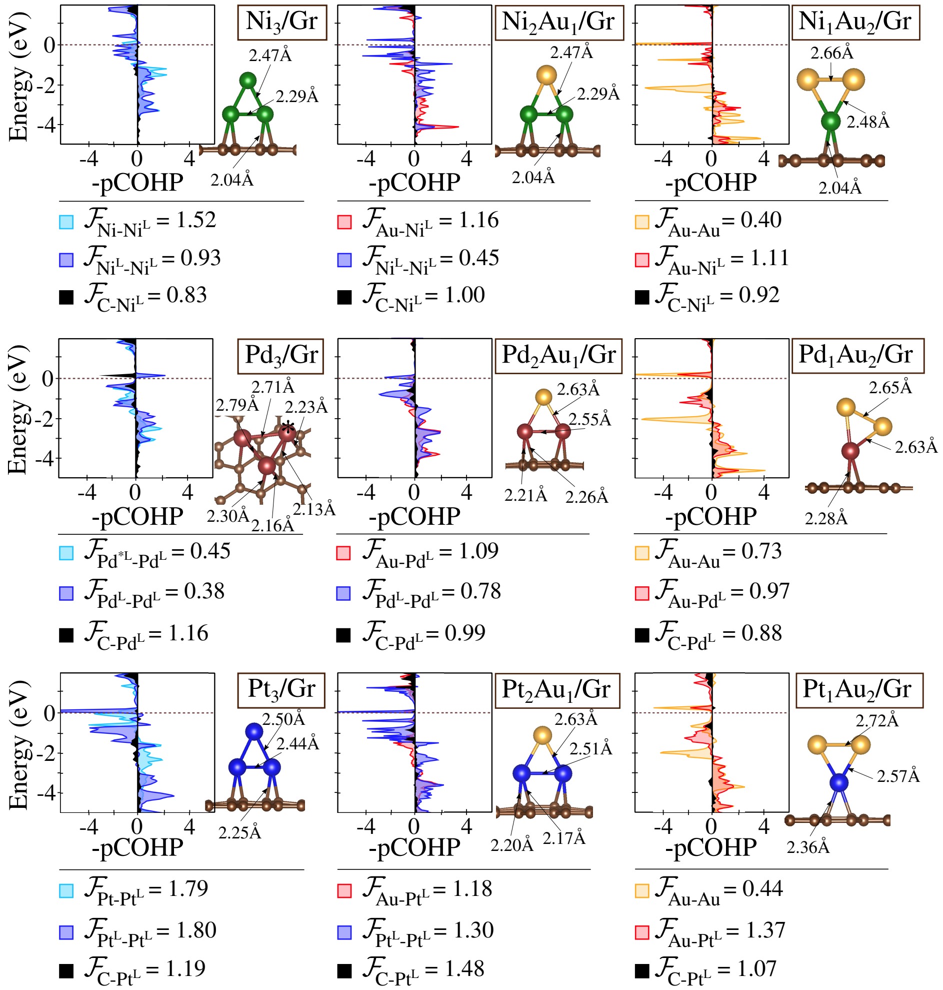

Figure S13 – Crystal Orbital Hamilton Population (COHP) analysis for unary and binary trimers (n = 3) clusters adsorbed on graphene (Gr) for M–M, M–Au, Au–Au, and C–M interactions, which $|\mathcal{F}_{\text{M-Au,Au-Au,M-M,C-M}}|$ as the covalent bond strength through the integration of COHP (ICOHP) values provided in eV and normalized by equivalent bonds. The ^{L} notation denotes the metal atoms bonded to Gr, while the asterisk (*) indicates configurations where a given element adopts a non-similar structural arrangement.

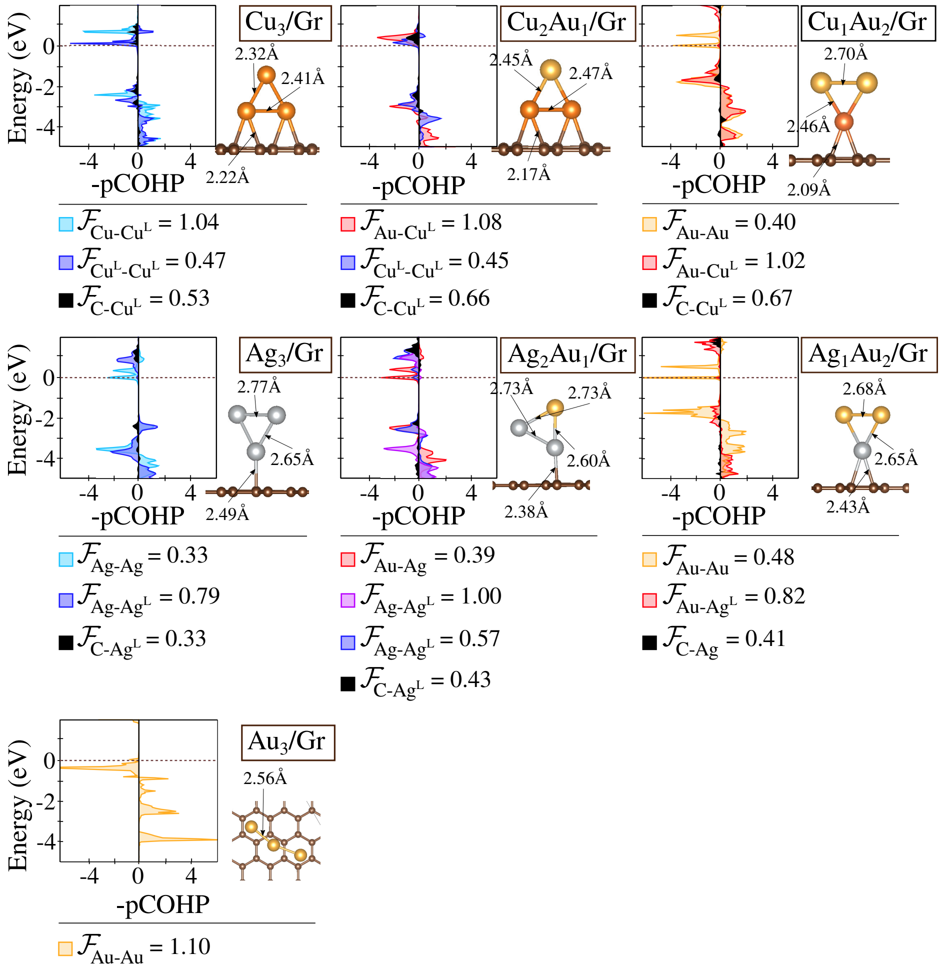

Figure S14 – Crystal Orbital Hamilton Population (COHP) analysis for unary and binary trimers (n = 3) clusters adsorbed on graphene (Gr) for M−M, M−Au, Au−Au, and C−M interactions, which $|\mathcal{F}_{\text{M-Au,Au-Au,M-M,C-M}}|$ as the covalent bond strength through the integration of COHP (ICOHP) values provided in eV and normalized by equivalent bonds. The ^{L} notation denotes the metal atoms bonded to Gr, while the asterisk (*) indicates configurations where a given element adopts a non-similar structural arrangement.

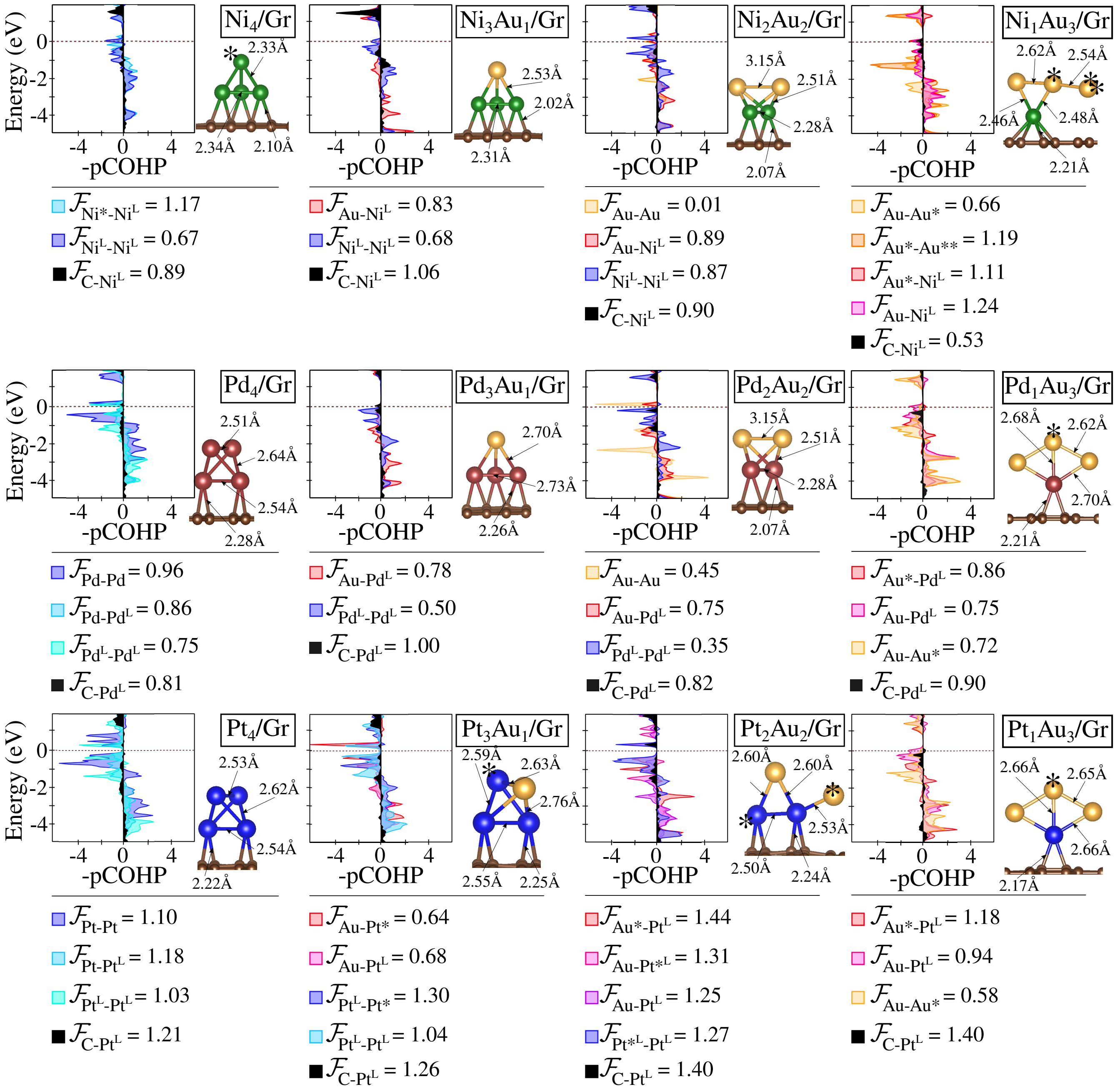

Figure S15 – Crystal Orbital Hamilton Population (COHP) analysis for unary and binary tetramers ($n = 4$) clusters adsorbed on graphene (Gr) for M−M, M−Au, Au−Au, and C−M interactions, which $|\mathcal{F}_{M\text{-}Au,Au\text{-}Au,M\text{-}M,C\text{-}M}|$ as the covalent bond strength through the integration of COHP (ICOHP) values provided in eV and normalized by equivalent bonds. The ^{L} notation denotes the metal atoms bonded to Gr, while the asterisk (*) indicates configurations where a given element adopts a non-similar structural arrangement.

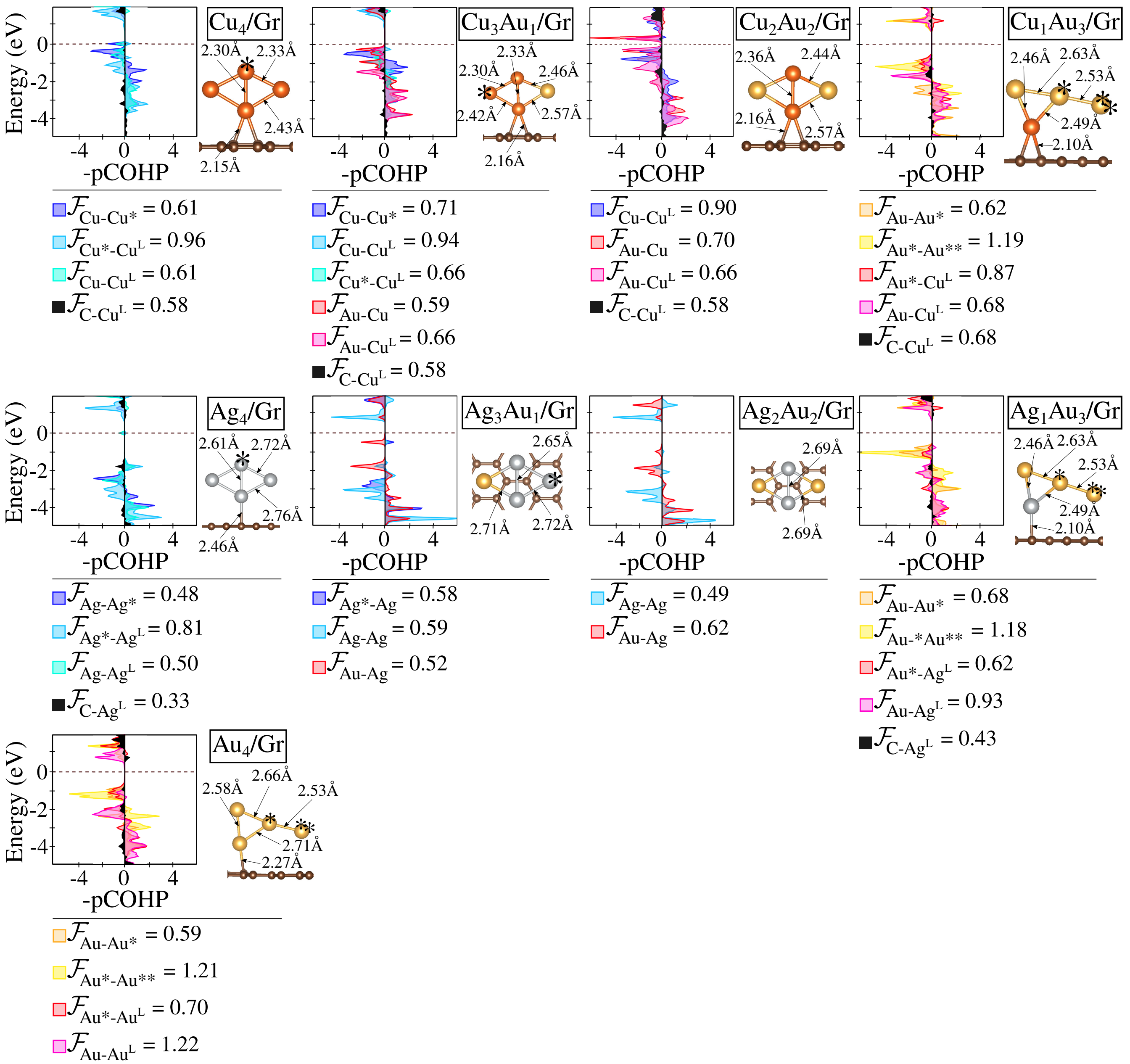

Figure S16 – Crystal Orbital Hamilton Population (COHP) analysis for unary and binary tetramers (n = 4) clusters adsorbed on graphene (Gr) for M−M, M−Au, Au−Au, and C−M interactions, which $|\mathcal{F}_{\text{M-Au,Au-Au,M-M,C-M}}|$ as the covalent bond strength through the integration of COHP (ICOHP) values provided in eV and normalized by equivalent bonds. The ^{L} notation denotes the metal atoms bonded to Gr, while the asterisk (*) indicates configurations where a given element adopts a non-similar structural arrangement.

7 Catalytic potential

A literature survey identified several studies that provide information on the electronic structure, particularly the d-band center (ε_d), of novel nanocatalysts developed with high efficiency for hydrogen evolution reactions (HER), oxygen evolution reactions (OER), and oxygen reduction reactions (ORR). Based on these ε_d values, we estimate an optimal range of −1 to −2 eV to evaluate the catalytic potential of the alloy systems investigated in this study.

Catalysts	Reaction type	ε_d (eV)
Ru clusters (~1.0 to 3.0 nm)[17]	HER	−1.60 to −1.80
Pt−Ni@Pt_D/G (nanoparticles with ~3.0 to 5.0 nm)[18]	ORR	−1.90 to −1.14
Mo−WC@NCS (nanoparticles with ~5.0 nm)[19]	HER	−1.22
MoC/Co–N–C–600[20]	OER	−1.90
VFe–doped nickel sulfide[21]	HER and OER	−0.97
CoNi nanoparticles (10.8±0.5 nm)[22]	OER	−2.08

Table S10 – Referenced catalysts involved in HER, OER, and ORR are presented along with their respective d-band centers (ε_d).

8 Densitiy of states (DOS)

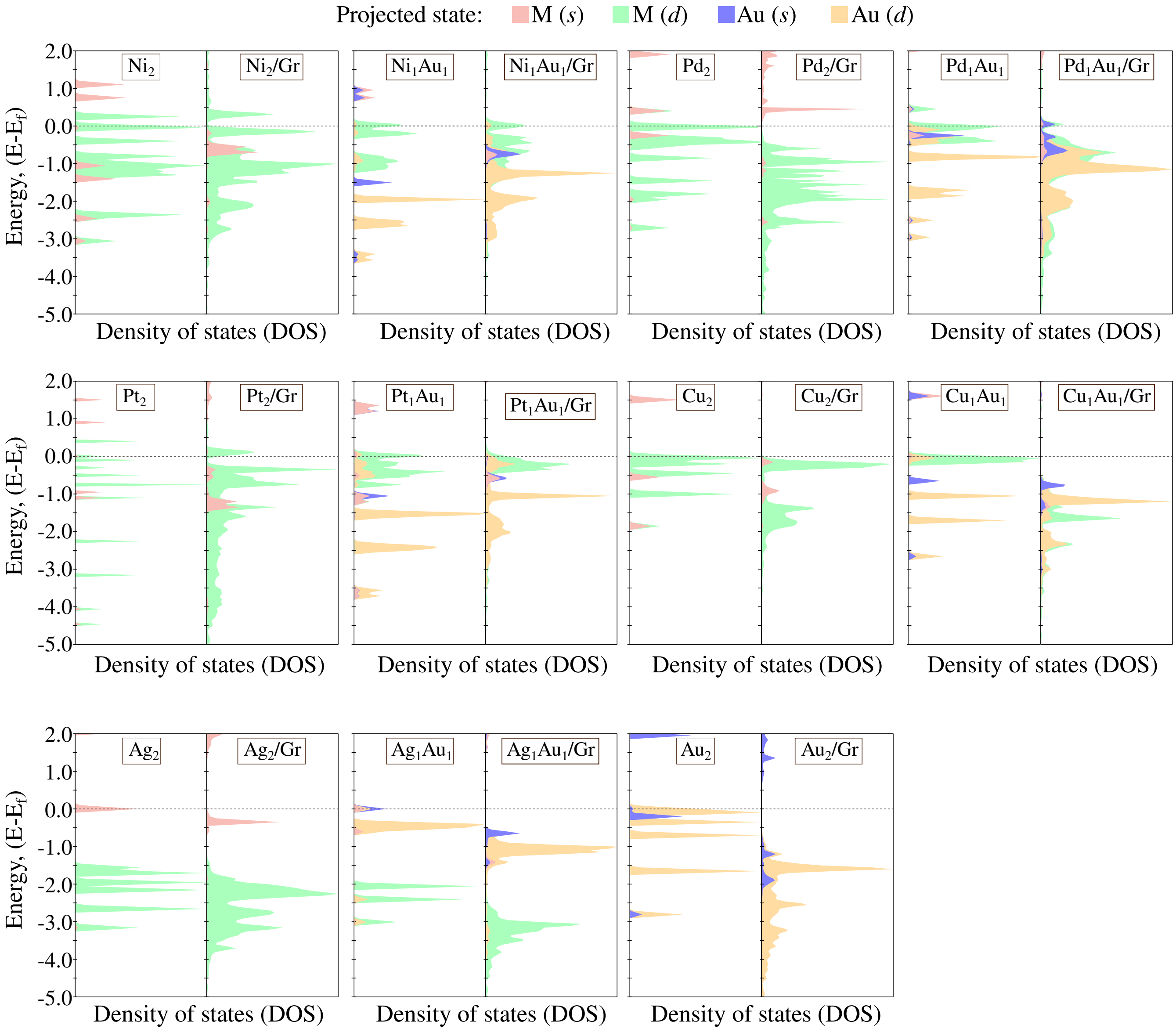

Figure S17 – Projected density of states (DOS) for the *s*- and *d*-states of M and Au atoms in unary and binary clusters, analyzed under vacuum conditions and upon adsorption on graphene.

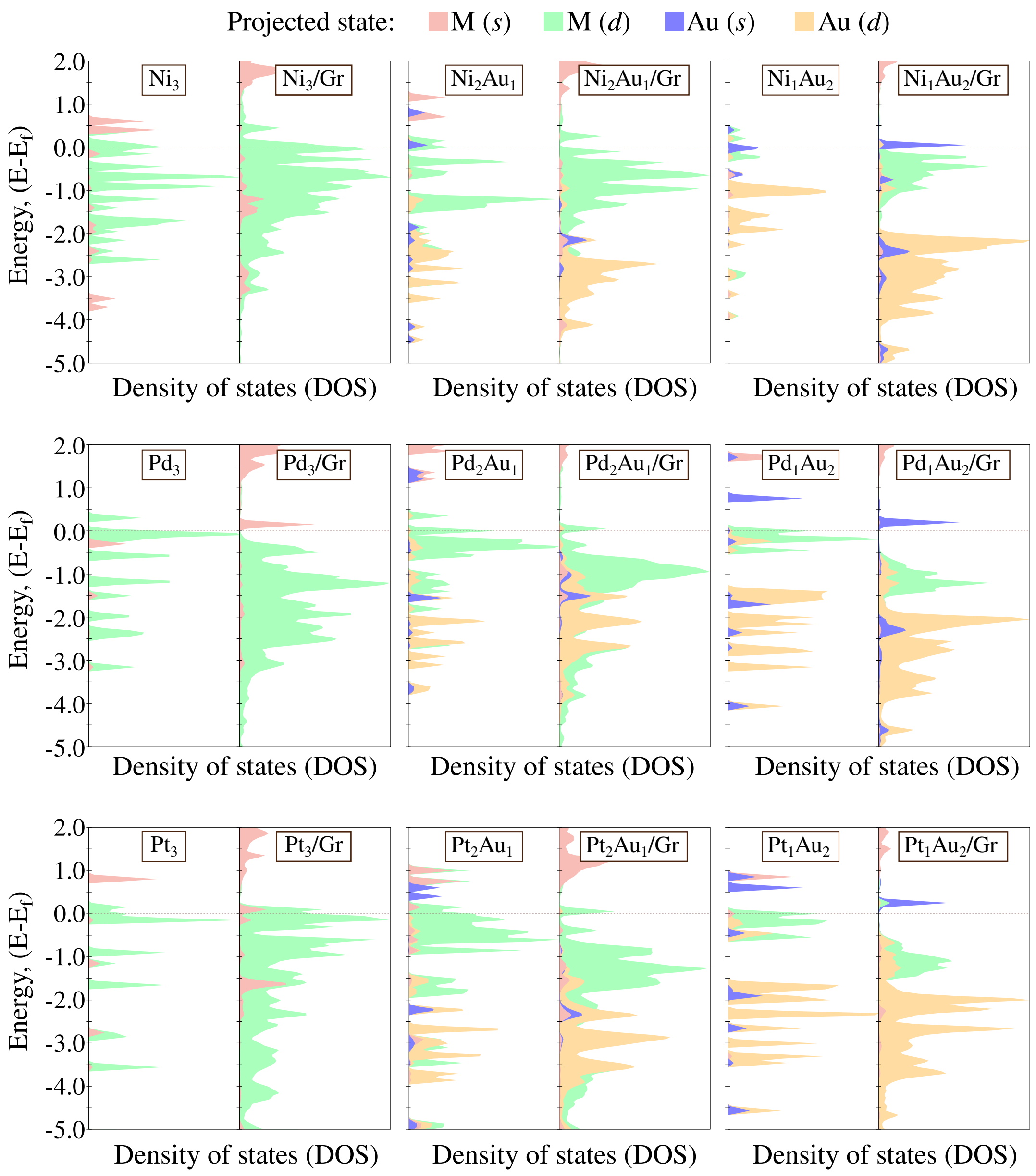

Figure S18 – Projected density of states (DOS) for the *s*- and *d*-states of M and Au atoms in unary and binary clusters, analyzed under vacuum conditions and upon adsorption on graphene.

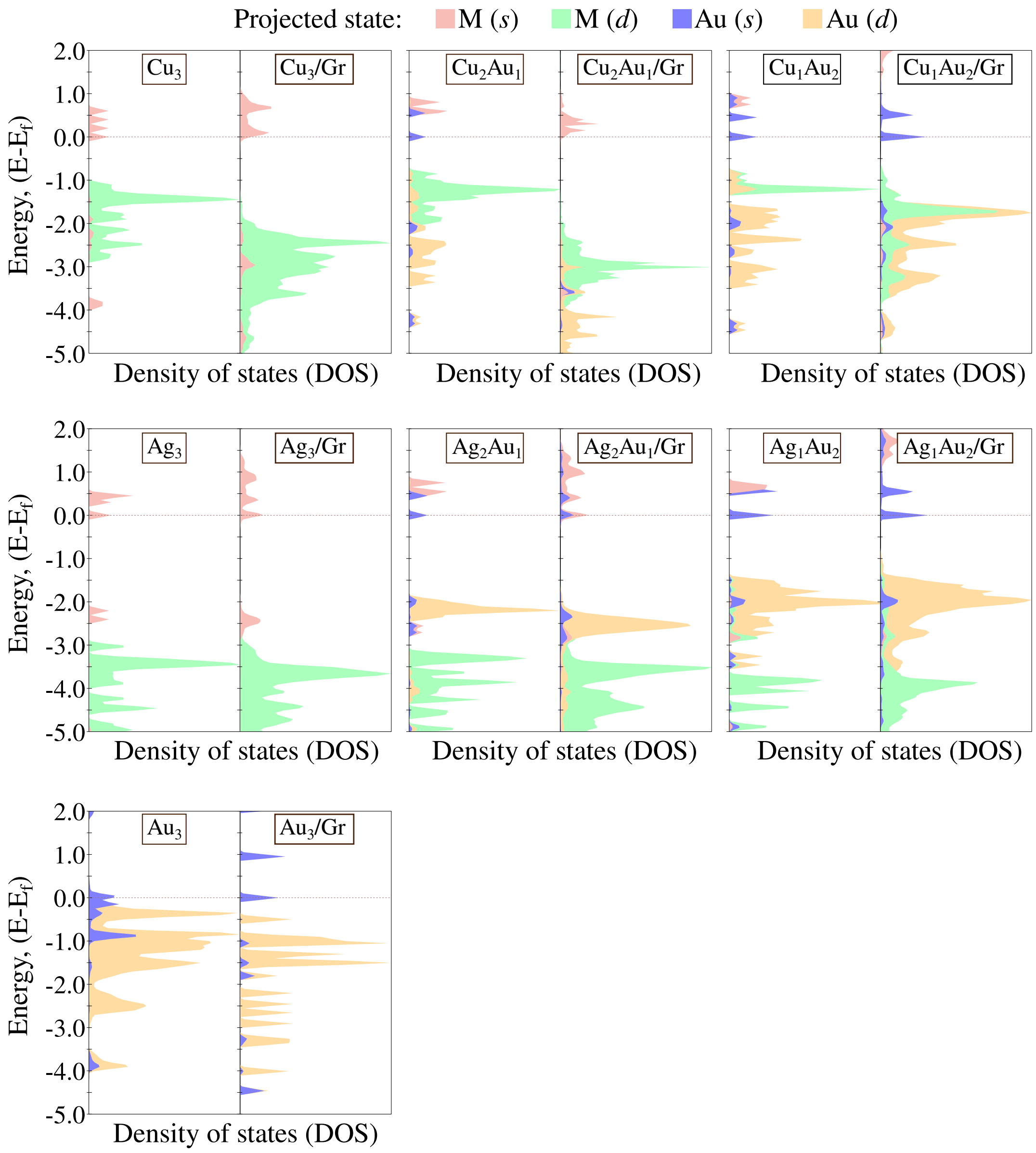

Figure S19 – Projected density of states (DOS) for the *s*- and *d*-states of M and Au atoms in unary and binary clusters, analyzed under vacuum conditions and upon adsorption on graphene.

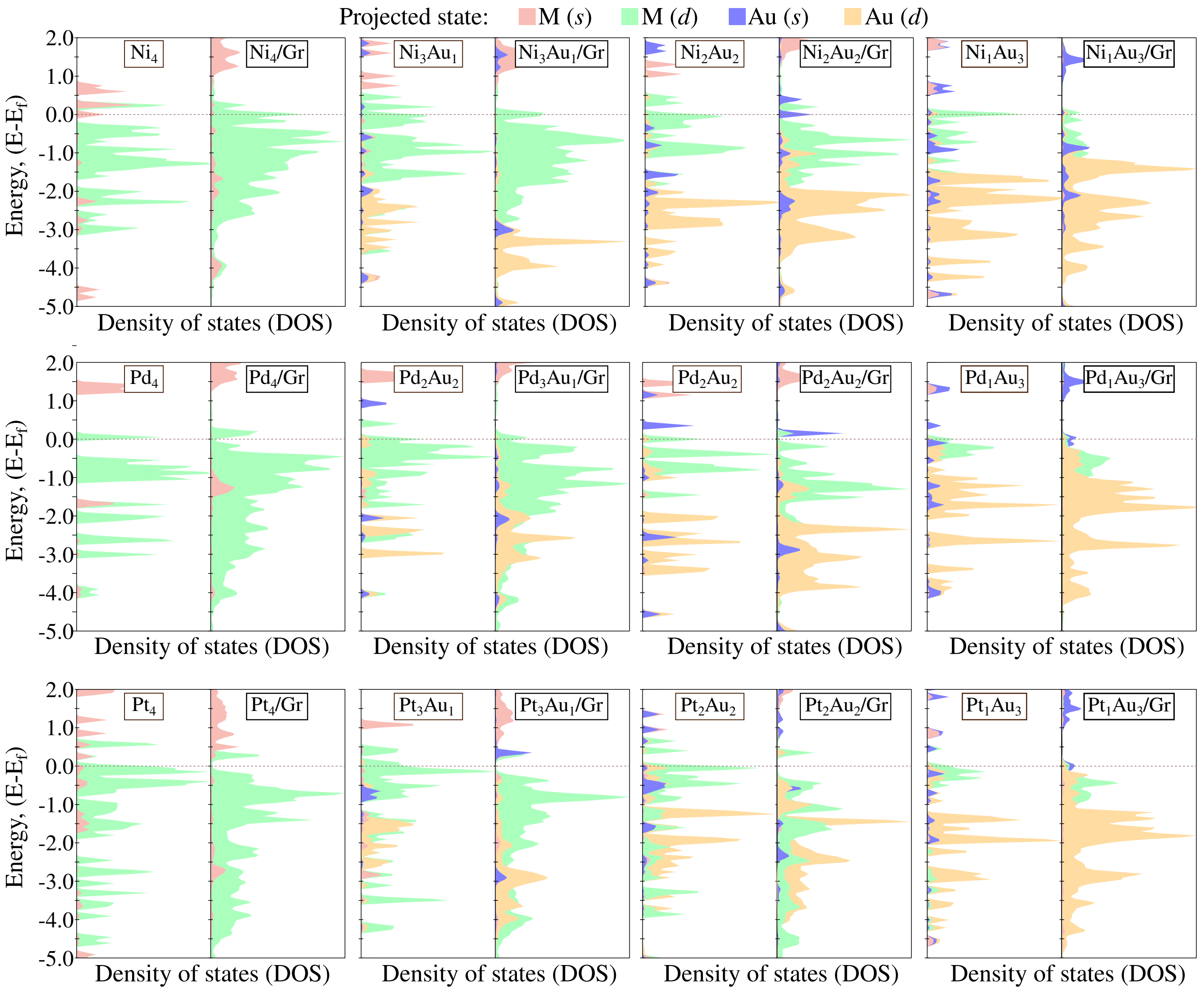

Figure S20 – Projected density of states (DOS) for the s- and d-states of M and Au atoms in unary and binary clusters, analyzed under vacuum conditions and upon adsorption on graphene.

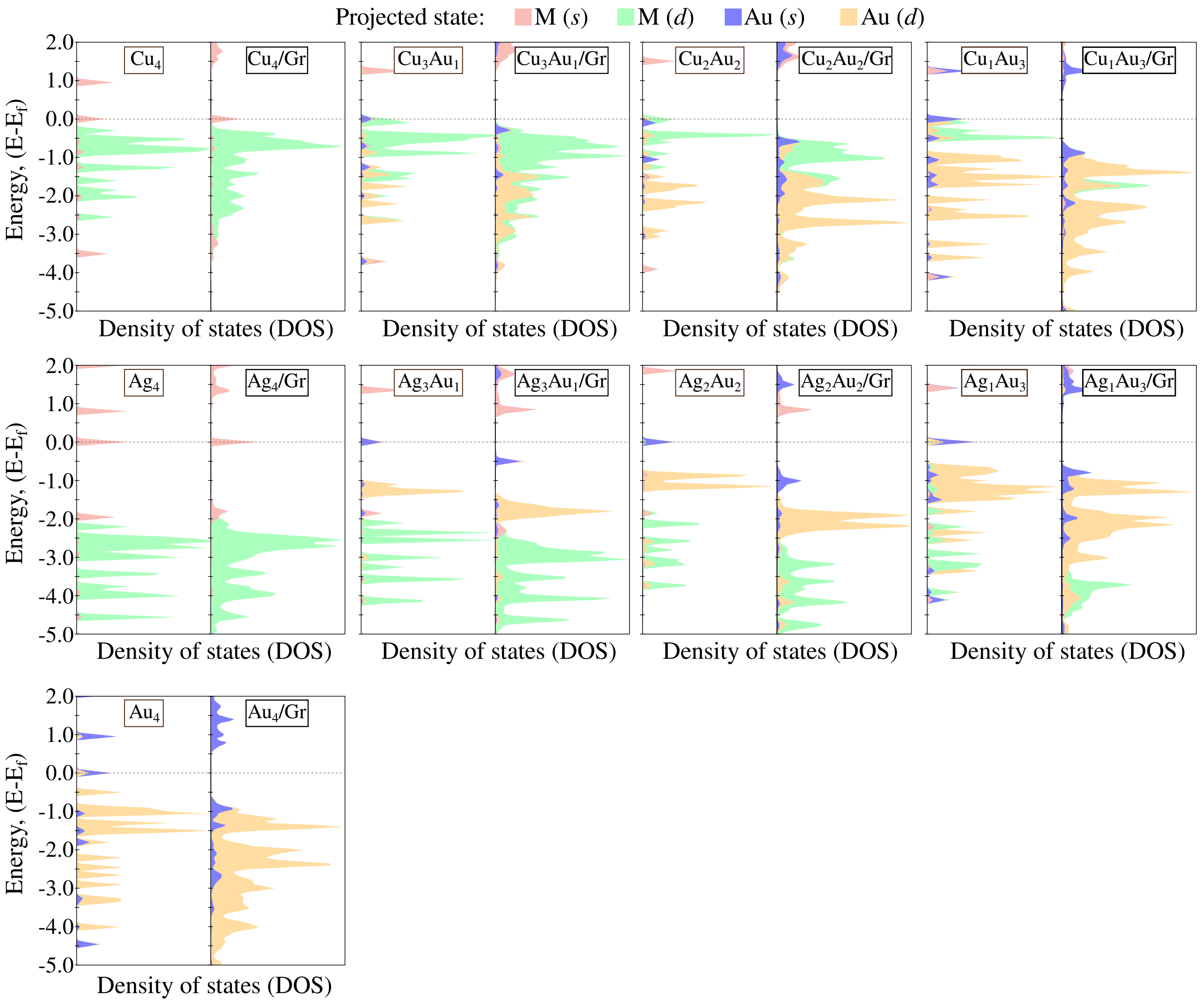

Figure S21 – Projected density of states (DOS) for the *s*- and *d*-states of M and Au atoms in unary and binary clusters, analyzed under vacuum conditions and upon adsorption on graphene.

9 Magnetic moment

Magnetic moment (μ_B)								
$n = 2$			$n = 3$			$n = 4$		
	vac.	ads.		vac.	ads.		vac.	ads.
Ni_1Au_1	1	1	Ni_1Au_2	2	0	Ni_1Au_3	1	1
Ni_2	2	2	Ni_2Au_1	3	1	Ni_2Au_2	2	2
			Ni_3	2	2	Ni_3Au_1	3	1
Pd_1Au_1	1	1				Ni_4	4	2
Pd_2	2	0	Pd_1Au_2	0	0			
			Pd_2Au_1	1	1	Pd_1Au_3	1	1
Pt_1Au_1	1	0	Pd_3	0	0	Pd_2Au_2	0	0
Pt_2	2	2				Pd_3Au_1	1	1
			Pt_1Au_2	0	0	Pd_4	2	2
Cu_1Au_1	0	0	Pt_2Au_1	1	1			
Cu_2	0	0	Pt_3	0	0	Pt_1Au_3	1	1
						Pt_2Au_2	2	0
Ag_1Au_1	0	0	Cu_1Au_2	1	1	Pt_3Au_1	1	1
Ag_2	0	0	Cu_2Au_1	1	0	Pt_4	2	2
			Cu_3	1	0			
Au_2	0	0				Cu_1Au_3	0	0
			Ag_1Au_2	1	1	Cu_2Au_2	0	0
			Ag_2Au_1	1	1	Cu_3Au_1	0	0
			Ag_3	1	1	Cu_4	0	0
			Au_3	1	1	Ag_1Au_3	0	0
						Ag_2Au_2	0	0
						Ag_3Au_1	0	0
						Au_4	0	0

Table S11 – Magnetic moment provide in Bohr magneton (μ_B) for all unary and binary dimers (n = 2), trimers (n = 3), and tetramers (n = 4) in both vacuum (vac.) and adsorbed on graphene (ads.) conditions.

References

1. Jin, C. *et al.* Adsorption of transition-metal clusters on graphene and N–doped graphene: a DFT study. *Langmuir* **38**, 3694–3710, DOI: https://doi.org/10.1021/acs.langmuir.1c03187 (2022).
2. Rego, C. R., Tereshchuk, P., Oliveira, L. N. & Da Silva, J. L. Graphene-supported small transition-metal clusters: A density functional theory investigation within van der Waals corrections. *Phys. Rev. B* **95**, 235422, DOI: https://doi.org/10.1103/PhysRevB.95.235422 (2017).
3. Morse, M. *et al.* Spectroscopic studies of the jet-cooled nickel dimer. *J. Chem. Phys.* **80**, 5400–5405, DOI: http://dx.doi.org/10.1063/1.446646 (1984).
4. Gingerich, K. A. Experimental and predicted stability of diatomic metals and metallic clusters. *Faraday Symp. Chem. Soc.* **14**, 109–125, DOI: https://doi.org/10.1039/FS9801400109 (1980).
5. Gupta, S. K., Nappi, B. M. & Gingerich, K. A. Mass spectrometric study of the stabilities of the gaseous molecules diatomic platinum and platinum-yttrium. *Inorg. Chem.* **20**, 966–969, DOI: https://doi.org/10.1021/ic50218a004 (1981).
6. Feng, G., Ganduglia-Pirovano, M. V., Huo, C.-F. & Sauer, J. Hydrogen spillover to copper clusters on hydroxylated γ-Al_2O_3. *J. Phys. Chem. C* **122**, 18445–18455, DOI: https://doi.org/10.1021/acs.jpcc.8b03764 (2018).
7. Schissel, P. Dissociation energies of Cu_2, Ag_2, and Au_2. *J. Chem. Phys.* **26**, 1276–1280, DOI: https://doi.org/10.1063/1.1743507 (1957).
8. Huber, K. *Molecular spectra and molecular structure: IV. Constants of diatomic molecules* (Springer Science & Business Media, 2013). https://doi.org/10.1007/978-1-4757-0961-2.
9. Zhuo, H.-Y. *et al.* Theoretical understandings of graphene-based metal single-atom catalysts: stability and catalytic performance. *Chem. Rev.* **120**, 12315–12341, DOI: https://doi.org/10.1021/acs.chemrev.0c00818 (2020). https://pubs.acs.org/doi/full/10.1021/acs.chemrev.0c00818.
10. Sun, C. *et al.* Modulation of graphene and graphdiyne by metal$_n$ ($n = 1-5$) adsorption and nucleation and the effect on hydrogen evolution reaction. *Appl. Surf. Sci.* **580**, 152197, DOI: https://doi.org/10.1016/j.apsusc.2021.152197 (2022).
11. Wella, S. A., Hamamoto, Y., Morikawa, Y., Hamada, I. *et al.* Platinum single-atom adsorption on graphene: a density functional theory study. *Nanoscale Adv.* **1**, 1165–1174, DOI: https://doi.org/10.1039/C8NA00236C (2019).
12. Hoppe, R. The coordination number - an "inorganic chameleon". *Angew. Chem. Int. Ed.* **9**, 25–34, DOI: http://dx.doi.org/10.1002/anie.197000251 (1970).
13. Hoppe, R. Effective coordination numbers (ECoN) and mean active fictive ionic radii (MEFIR). *Z. Kristallogr.* **150**, 23–52, DOI: http://dx.doi.org/10.1524/zkri.1979.150.1-4.23 (1979).
14. Manz, T. A. & Limas, N. G. Introducing DDEC6 atomic population analysis: Part 1. charge partitioning theory and methodology. *RSC Adv.* **6**, 47771–47801, DOI: https://doi.org/10.1039/D2RA90050E (2016).
15. Bader, R. F. W. *Atoms in Molecules: A Quantum Theory*. International Series of Monographs on Chemistry (Clarendon Press, 1994). https://doi.org/10.1002/ange.19921041040.
16. Dronskowski, R. & Blöchl, P. E. Crystal orbital hamilton populations (COHP): energy-resolved visualization of chemical bonding in solids based on density-functional calculation. *J. Phys. Chem.* **97**, 8617–8624, DOI: https://doi.org/10.1021/j100135a014 (1993).
17. Hu, Q. *et al.* Subnanometric Ru clusters with upshifted *d*–band center improve performance for alkaline hydrogen evolution reaction. *Nat. Commun.* **13**, 3958, DOI: https://doi.org/10.1038/s41467-022-31660-2 (2022).
18. Lyu, X. *et al.* Gradient-concentration design of stable core–shell nanostructure for acidic oxygen reduction electrocatalysis. *Adv. Mater.* **32**, 2003493, DOI: https://doi.org/10.1002/adma.202003493 (2020).
19. Wang, L. *et al.* Tuning *d*-band center of tungsten carbide via Mo doping for efficient hydrogen evolution and Zn−H_2O cell over a wide pH range. *Nano Energy* **74**, 104850, DOI: https://doi.org/10.1016/j.nanoen.2020.104850 (2020).
20. Liu, J., Guo, Y., Fu, X.-Z., Luo, J.-L. & Zhi, C. Strengthening absorption ability of Co−N−C as efficient bifunctional oxygen catalyst by modulating the *d* band center using MoC. *GEE* **8**, 459–469, DOI: https://doi.org/10.1016/j.gee.2021.05.008 (2023).
21. Zhou, J. *et al.* Precisely tailoring the *d*–band center of nickel sulfide for boosting overall water splitting. *Appl. Catal., B* **359**, 124461, DOI: https://doi.org/10.1016/j.apcatb.2024.124461 (2024).
22. Song, Q. *et al.* Enhanced electrocatalytic performance through body enrichment of Co–based bimetallic nanoparticles *in situ* embedded porous N–doped carbon spheres. *Small* **15**, 1903395, DOI: https://doi.org/10.1002/smll.201903395 (2019).
